# Near-Infrared and Shortwave Infrared Building Blocks for Activity-Based Sensors in Animals

**DOI:** 10.1021/acs.bioconjchem.6c00039

**Published:** 2026-06-02

**Authors:** Tianmiao Wang, Xinyuan Li, Lei Guo, Shang Jia

**Affiliations:** † Department of Chemistry and Biochemistry, 67206 Rutgers University—Newark, Newark, New Jersey 07102, United States; ‡ Department of Civil Engineering, 3341 University of Arkansas, Fayetteville, Arkansas 72701, United States

## Abstract

Activatable fluorescent probes operating in the near-infrared (NIR, 700–1000 nm) and shortwave-infrared (SWIR, also known as NIR-II, 1000–1700 nm) windows have become essential tools for bioimaging due to their deep tissue penetration, low background interference, and high signal-to-noise ratios. With growing numbers of such probes in the red-shifted windows developed for detection in animal models, this review summarizes how and what NIR and SWIR fluorophores can be coupled with selective triggers to create activity-based sensing NIR/NIR-II probes rather than listing probes based on their bioanalytes. To date, a variety of probes have been constructed this way, involving the modular combination of a fluorophore scaffold that defines photophysical properties, defined modification sites where caging groups are installed, and connection chemistry that links responsive moieties to the fluorophore core. Through representative examples, we illustrate how the same fluorophore can be adapted for diverse applications by varying the caging group, and how the same responsive chemistry can be implemented across different scaffolds by preserving the linkage type. This modular framework provides researchers with a practical design toolkit for constructing activatable probes for new biological targets.

## Graphical abstract

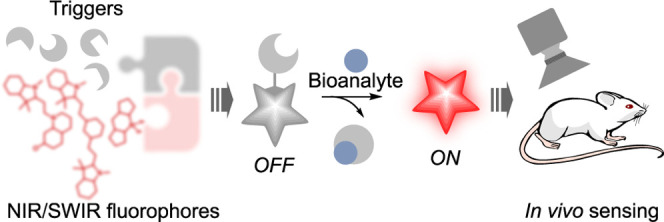

## 1 Introduction

Fluorescence imaging has emerged as an indispensable tool for visualizing biological processes with high spatial and temporal resolution in living systems.^1^ Among the diverse spectral windows available for bioimaging, the near-infrared (NIR, 700–1000 nm) and shortwave infrared (SWIR, also known as NIR-II, 1000–1700 nm) regions have attracted tremendous attention owing to their superior tissue penetration depth which reduced autofluorescence interference and minimized photon scattering compared to visible-light imaging in whole animal context.^2,3^ These intrinsic advantages position NIR/SWIR fluorescence imaging as a powerful modality for noninvasive visualization of deep-seated tissues, real-time monitoring of physiological and pathological processes, and image-guided surgical interventions.

Within the fluorescent imaging toolkit, activatable fluorescent probes, also termed “smart” or “turn-on” probes, represent a particularly attractive class of molecular sensors due to their ability to detect biological processes.^4–6^ Unlike “always-on” fluorophores that emit constitutively regardless of their biological environment, activatable probes remain dark in their native state and selectively generate fluorescence signals only under specific biological stimuli. This can be roughly categorized into two classes: Binding-Based Sensors (BBSs, Figure 1a), which rely on the reversible binding of the analyte and the probe. And, Activity-Based Sensors (ABSs, Figure 1b), where a chemoselective reaction elicits fluorescence turn-on of the fluorophore.^7–11^ Due to the dramatic structural change accompanying activation, ABS can be constructed modularly by combining a fluorophore that is subject to on/off modulation with structural change and a selective trigger for the biological process. In this context, the photophysical properties (i.e., wavelength and brightness after activation) are dictated by the fluorophore building block, whereas the trigger moiety defines the biological process for detection. As such, understanding how to effectively turn on and off the emission of a fluorophore through structural modification becomes critical in adapting biosensors for detection in different biological models.

**Figure 1:**
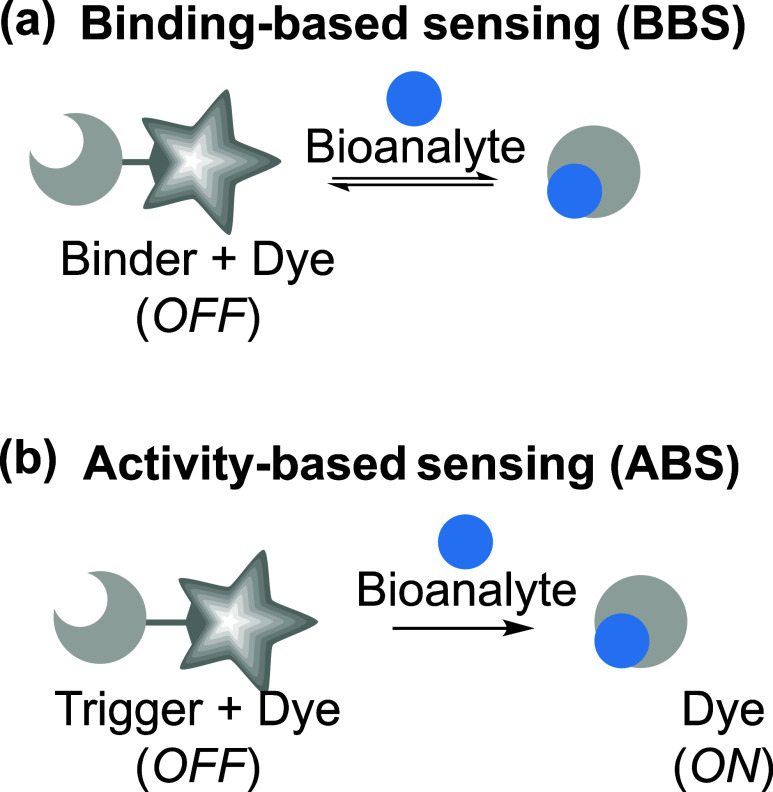
Approaches for designing fluorescent turn-on probes. (a) Binding-based sensing approach. (b) Activity-based sensing approach.

Similar to the track of always-on dyes in bioimaging, fluorophores for incorporation into sensors also have various approaches. Specifically, fluorescent materials, such as inorganic nanoparticles^12,13^ and conjugated polymers,^14,15^ can be made responsive toward biological stimuli by quenching or electron transfer mechanisms upon surface modification Moreover, molecular fluorophores can be structurally modified to have change of aggregation states upon detection of bioanalytes, which elicits fluorescent readout, such as in scaffolds capable of aggregation-induced emission.^16,17^ While benefiting from comparatively higher brightness and photostability over the fully soluble molecular fluorophores, these materials-based approaches can lack defined structure and often associate with long-term toxicity owing to their difficulty of clearance from animals. We hereby confine our discussion to molecular-based fluorophores capable of modular construction into activity-based sensors in this Review.

Compared to their visible counterparts, NIR/SWIR sensors enable the detection of biological processes in vivo within complete physiological context, with direct implications for diagnostics and therapeutic monitoring.^18^ However, despite the explosive growth in biosensor development in the NIR/SWIR regions, these probes remain comparatively less established than visible-light sensors, which are useful for cellular detection but difficult for imaging in animals. Following the modular construction principle of ABS sensors, a major bottleneck in developing NIR/SWIR sensors is the availability of robust and reliable redshifted-fluorophores amenable to on/off modulation through attachment of selective triggers. Although not as established as well-known visible building blocks such as fluorescein or rhodamine derivatives, several NIR fluorophore scaffolds and respective connection approaches have demonstrated effectiveness as building blocks to construct NIR-ABS sensors, and extension toward SWIR for clearer and sharper detection in animals is underway. This review focuses on reported NIR/SWIR fluorophore scaffolds that can be used in a plug-and-play manner as building blocks for constructing ABS sensors for in vivo detection. We systematically examine three major platforms that have been successfully developed as ABS building blocks, including BODIPY, polymethine, and hemicyanine dyes, the connection chemistries employed to install responsive triggers, and the fluorescence activation mechanisms that govern signal generation. These building blocks have been coupled with a series of triggers, most of which were initially developed with visible fluorophores, enabling detection of reactive oxygen, nitrogen, and sulfur species, biothiols, and visualization of enzymatic activities in animal models. Rather than organizing probes by detection targets, we adopt a scaffold-centric perspective that emphasizes how probes are constructed: which sites are modified, what linkage types connect triggers to fluorophores, and what electronic or structural changes activate fluorescence. We hope this NIR/SWIR fluorophore and modification-focused review can provide reference for translating analyte detection triggers into ABS sensors designed for in vivo imaging. Readers seeking specific probes for particular analytes can refer to existing reviews with analyte-centric frameworks.^19–28^

## 2 BODIPY and Its Derivatives as Platforms for NIR (and NIR-II) Activatable Probes

BODIPY (4,4-difluoro-4-bora-3a,4a-diaza-*s*-indacene, Figure 2) derivatives were first discovered by Treibs and Kreuzer in 1968. These fluorophores exhibit exceptional photostability, narrow emission bands, high quantum yields, and favorable molar extinction coefficients, making them attractive scaffolds for fluorescent probe development.^36,37^ However, the intrinsic hydrophobicity of the BODIPY core often leads to poor solubility in aqueous media, which presents challenges for biological applications. Moving beyond the unsubstituted BODIPY fluorophore, which emits around 505 nm, the 3 or 5-positions provide handles for π conjugation extension, enabling bathochromic shifts into the NIR and even SWIR windows.^38,39^ Despite the lack of apparent hydroxyl or amino handle in the pristine BODIPY structure for trigger attachment to modulate its fluorescence on/off, substitutions can be meticulously introduced at the 8- or 3-site, which can subsequently be functionalized with triggers for designing ABS sensors. A summary of the probes discussed in this section is provided in Table S1.

**Figure 2:**
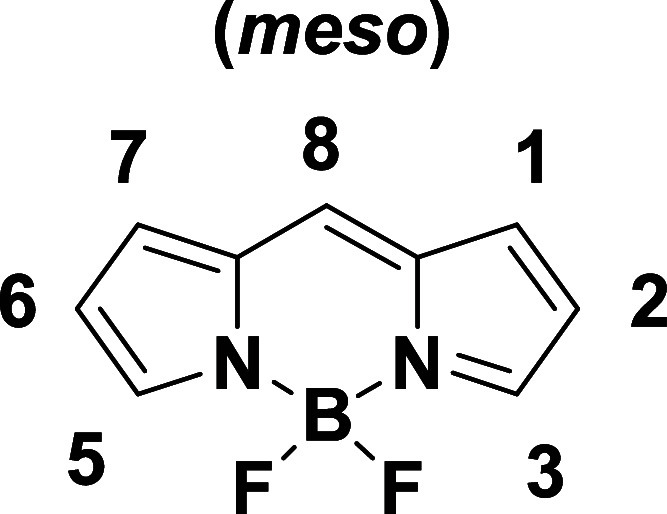
Structure of the BODIPY dye scaffold.

### 2.1 3-Thiol-Functionalized BODIPY Platform: ICT-Based Activation through Sulfur Conjugation

The photophysical behavior of 3-mercapto-substituted BODIPYs is governed by the oxidation state and substitution pattern of the sulfur atom, establishing a robust paradigm for activatable probe design. In particular, the free thiol acts as a strong electron-donating group, furnishing an effective push–pull system with a strong bathochromic shift and higher brightness. In contrast, masking the 3-mercapto group as a thioether reduces its electron-donating characteristics, producing a hypsochromically shifted absorption band and a weakly emissive OFF state. This fluorescence change can be leveraged toward sensor design with the help of a self-immolative benzyl linker on the thioether,^40,41^ converting the release of amino or hydroxyl groups caged in selective triggers into free mercapto group on the BODIPY fluorophore via 1,6-elimination. Regeneration of the free 3-mercapto substituent restores the original electronic structure of the fluorophore, shifting the absorption back to longer wavelengths and recovering fluorescence to produce the ON state (Figure 3).

**Figure 3:**
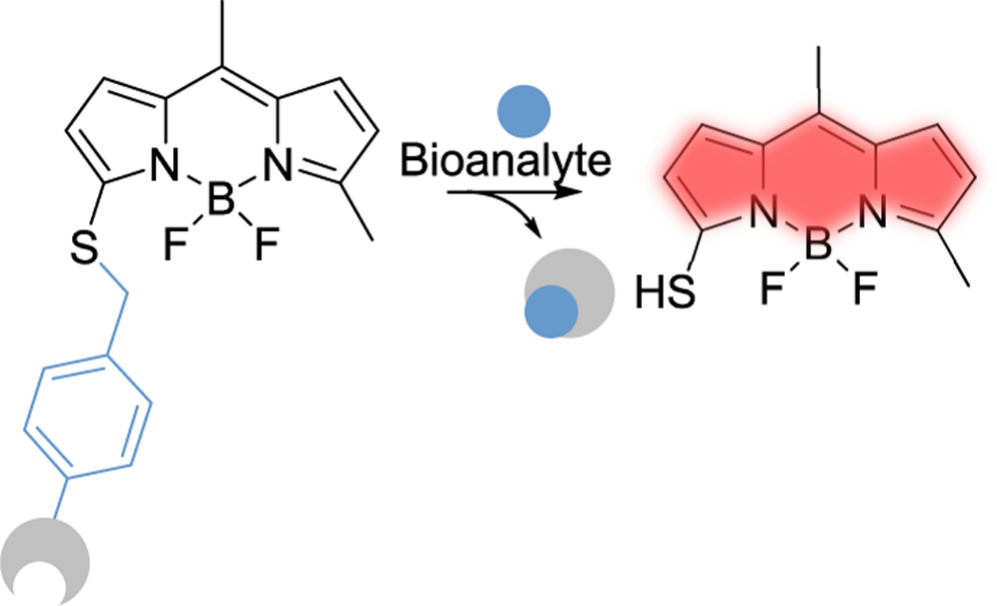
Scheme of 3-Mercapto-functionalized BODIPY in ABS sensor design.

The first report of sulfur substitution to modulate BODIPY photophysics was established by Yang and co-workers in 2012.^42^ In their design, a 3-chlorinated BODIPY served as the reactive scaffold, where the chlorine atom can be displaced by thiol on glutathione (GSH) through S_N_Ar substitution, resulting with thioether in place of –Cl and a fluorescence red-shift. This connection is further expanded into a fluorophore platform for ABS probes by Zhao and co-workers by coupling thiol-functionalized BODIPY to cleavable substrates via a self-immolative benzyl thioether linker (Figure 4a).^29^ In this modular architecture, the thiol of BODIPY is connected to a para-substituted benzyl group through a thioether bond, with the cleavable trigger installed at the *para*-position of the benzyl ring. This design creates a two-stage activation pathway: the trigger moiety first undergoes transformation to generate an electron-donating group (amine, phenol, or alkoxide) at the para-position, which subsequently initiates spontaneous 1,6-elimination to release the free mercapto group on -BODIPY. In the caged state, the benzyl thioether linkage reduces the electron-donating ability of the sulfur atom, confining absorption and emission to the visible region. Upon linker fragmentation, the liberated thiol restores strong electron-donating character, shifting emission into the NIR region through intramolecular charge transfer (ICT) in the excited state with large Stokes shifts. A key design advantage of this platform lies in the tunability of emission wavelength: by varying the electron-withdrawing acceptor conjugated at the 2-position through a vinylene bridge, the authors demonstrated that an imidazolone unit affords NIR-I emission at 725 nm with a 50 nm Stokes shift, while installation of a benz­[*e*]­indolium acceptor extends emission to 900 nm in the NIR-II window with a 170 nm Stokes shift. Accordingly, the authors prepared probe **1** (NTR-InD) for the detection of nitroreductase activity on cancer cells, which exhibits a 316-fold turn on in vitro experiment (Figure 4b) and successfully visualized the A549 tumor in a mouse xenograft model (Figure 4c). These probes can be made useful for the detection of specific cancer types and at the same time established that trigger selection and wavelength tuning can be optimized independently within the same modular framework.

**Figure 4:**
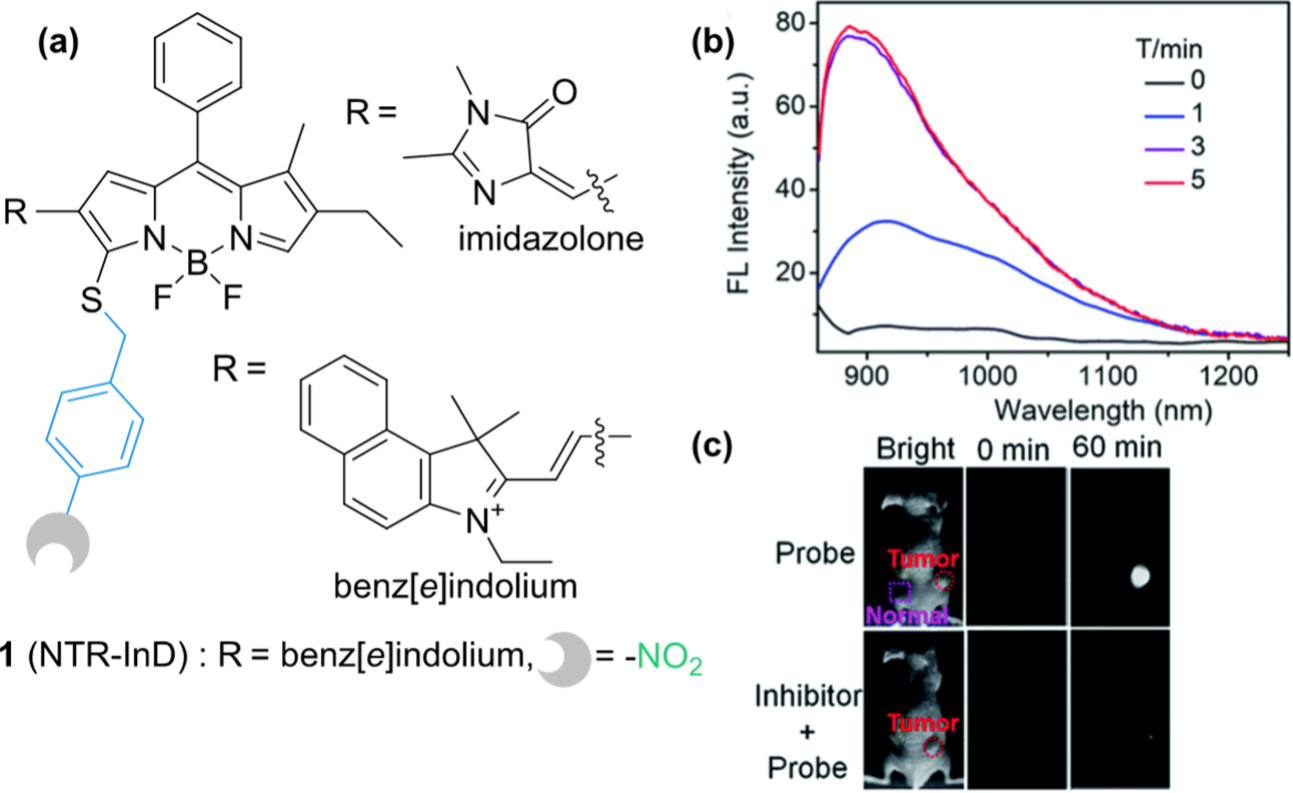
Thiol-functionalized BODIPY platforms for NIR/SWIR ABS sensors. (a) Modular design of 3-mercapto-BODIPY probes. (b) NIR-II fluorescence turn-on (λ_ex_ = 730 nm) of **1** (NTR-InD) upon addition of NTR. (c) In vivo NIR-II fluorescence imaging of nitroreductase activity in A549 tumor-bearing mice using NTR-InD at 0 and 60 min postinjection, without or with the dicoumarol nitroreductase inhibitor. Panel (b) and (c) adapted with permission from ref 29 Copyright 2019 The Royal Society of Chemistry.

Building upon this self-immolative benzyl thioether linker architecture, Gu and co-workers further demonstrated the platform versatility by synthesizing probes with different electron-withdrawing acceptors at the 2-position while preserving identical activation mechanisms. Two representative designs illustrate how this modification can affect emission wavelength: **2** (BOD-K-βGal) incorporates 1,2-dimethyl-1*H*-imidazol-5­(4*H*)-one as a moderate acceptor, whereas **3** (BOD-M-βGal) employs the stronger 3-ethyl-1,1,2-trimethyl-1H-benz­[e]­indolium unit to access the NIR-II window with excitation at 723 nm and emission at 853 nm (Figure 5a).^30^ Further extending this design principle, **4** (BOD-II-NAG-NP) conjugates 3-ethyl-2-methylbenzo­[*d*]­thiazol-3-ium at the 3-position through Knoevenagel condensation, pushing emission to approximately 1000 nm upon activation.^31^ In both cases, the core activation mechanism remains unchanged: cleavage of the trigger moiety generates a phenolate intermediate that initiates 1,6-elimination to release the fluorescent thiol-BODIPY. To address water solubility limitations for in vivo applications, BOD-II-NAG-NP was encapsulated into a mPEG-DSPE polymer matrix and was utilized to detect *N*-acetyl-β-D-glucosaminidase (NAG) in diabetic nephropathy group (Figure 5b). Such a design is particularly useful for interrogating disease-associated renal enzyme activity in vivo, as exemplified here in diabetic nephropathy. Furthermore, Liu and co-workers systematically extended the conjugation at the 2- and 5-position, which are common positions for red-shifting wavelength of BODIPY dyes, furnishing a series of red-shifted the emission fluorophores (WH-1 to WH-4, Figure 5c).^32^ These probes can be coupled with a 4-nitrobenzenethiol group as a trigger for H_2_S detection, which functions simultaneously as a fluorescence quencher and a displaceable leaving group. Following these design strategies, a series of selective triggers have been attached to this scaffold to construct NIR to SWIR fluorophores. Besides, a handful of mercapto-releasing triggers for H_2_S can be directly attached, such as 4-Nitrothiophenol and phenylselenide for H_2_S.^43^

**Figure 5:**
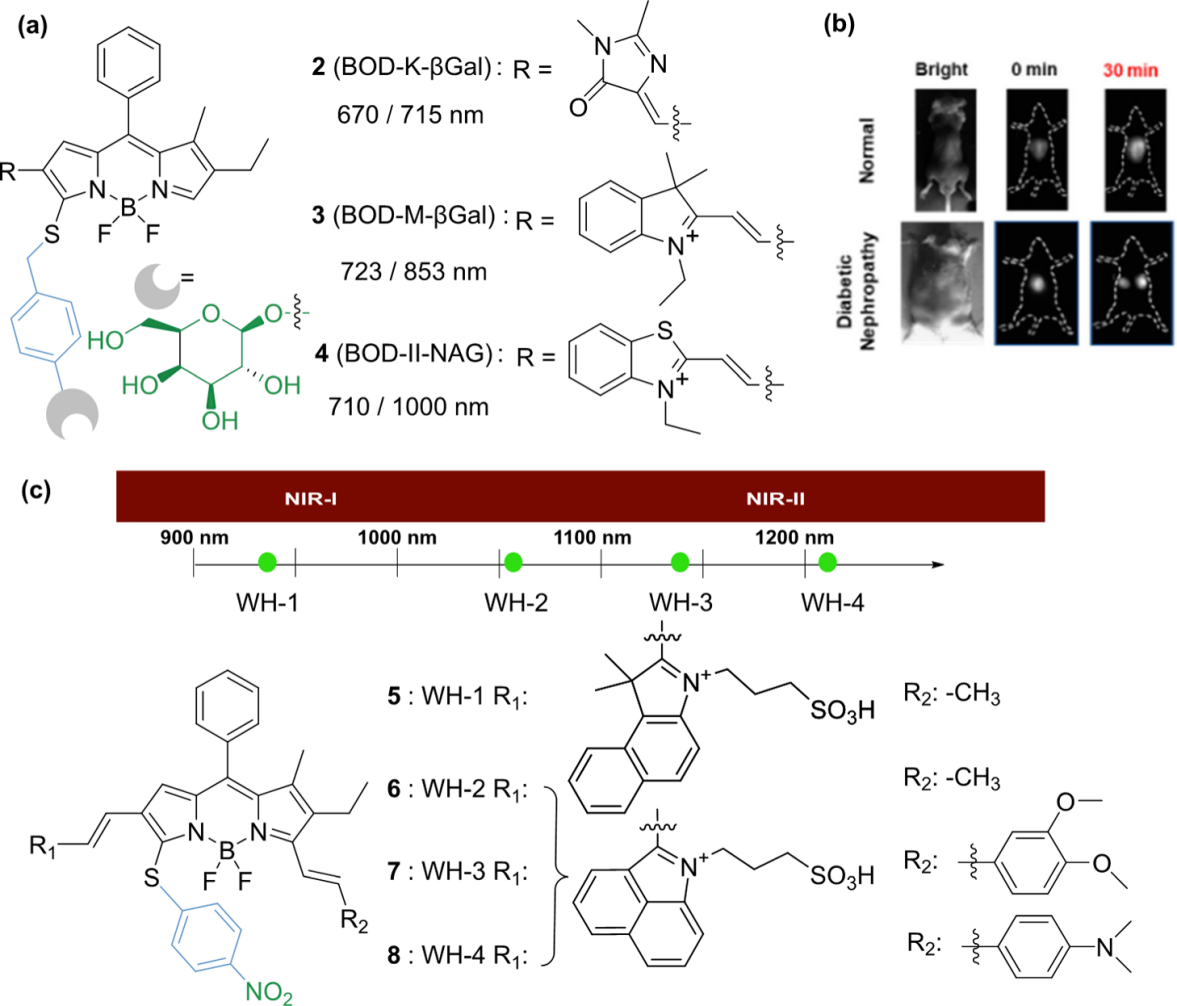
Thiol-functionalized BODIPY platforms with tunable NIR/SWIR emission. (a) Chemical structures of 3-mercapto-BODIPY probes with a β-d-galactose trigger and varying electron-withdrawing acceptors at the 2-position. Absorption and emission maxima are listed below each structure. (b) In vivo SWIR fluorescence imaging of NAG activity in normal versus diabetic nephropathy mouse models at 0 and 30 min postinjection using BOD-II-NAG-NP. Adapted with permission from ref 31 Copyright 2020 The Royal Society of Chemistry. (c) Wavelength tunability of thiol-BODIPY probes through systematic conjugation extension at the 5-position for H_2_S detection: 5 (WH-1), 6 (WH-2), 7 (WH-3), and 8 (WH-4) with emission maxima spanning from NIR-I (∼900 nm) to NIR-II (>1200 nm).

### 2.2 Meso-Benzylpyridinium on BODIPY: dPeT Quenching with Self-Immolative Release

Another general method for modulating BODIPY fluorescence is through the introduction of a pyridium moiety at the *meso*-position,^44^ which is connected to selective triggers through a self-immolative quinone methide linker. In the OFF state, the cationic pyridinium unit acts as a strong electron acceptor and lowers the HOMO energy of the substituent, enabling donor-excited photoinduced electron transfer (dPeT)-based quenching of its fluorescence. Upon analyte detection, the selective trigger releases a phenol intermediate that undergoes 1,6-elimination to convert the pyridinium into a neutral pyridyl species. This transformation weakens the electron-accepting ability of the meso substituent, suppresses the dPeT quenching pathway, and restores the emissive ON state of the BODIPY fluorophore (Figure 6).

**Figure 6:**
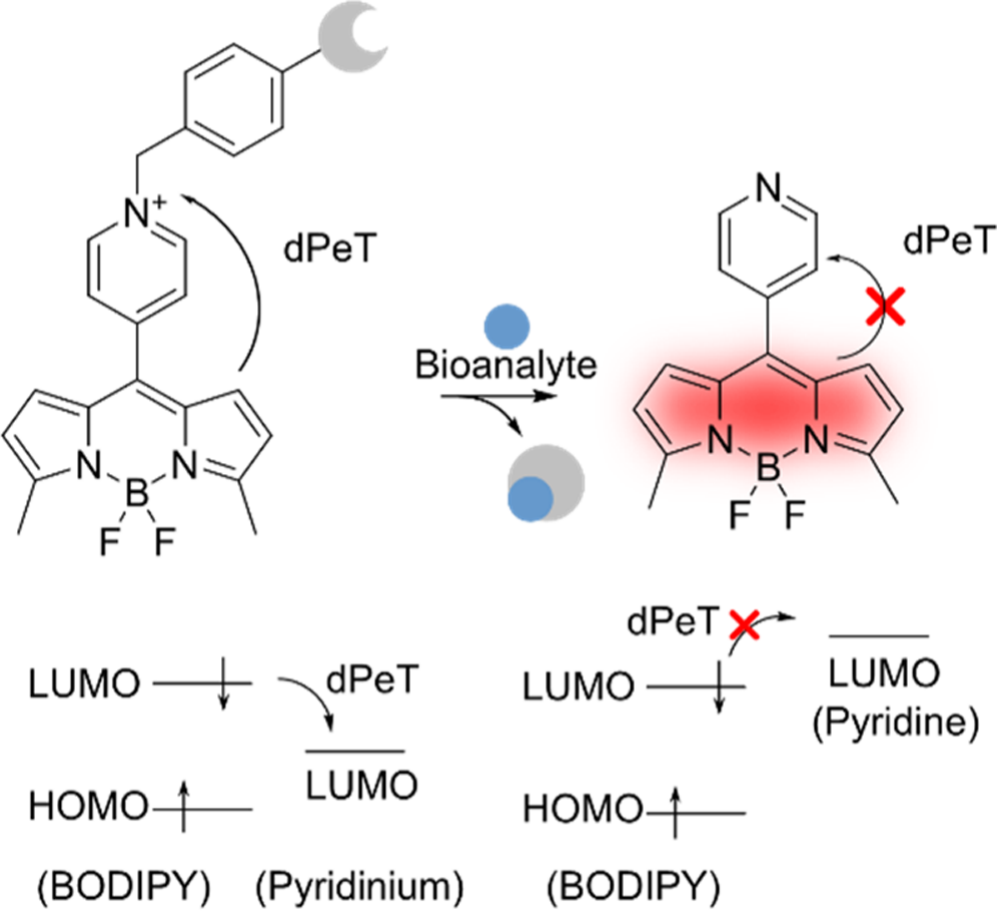
Mechanism of *meso*-pyridinium-based BODIPY ABS sensors utilizing dPeT quenching. Bottom: simplified molecular orbital diagrams illustrating the dPeT quenching mechanism (left) and its inhibition upon trigger cleavage (right).

Qiao and co-workers first established this cationic platform by conjugating 4-(bromomethyl)­phenylboronic acid to the *meso*-(4-pyridyl) on a visible fluorophore BODIPY to form the pyridinium salt through *N*-alkylation **9** (Figure 7a).^33^ The boronic acid functions as a selective trigger for hypochlorite, freeing the neutral pyridyl group after analyte reaction and linker elimination. The probe shows strong fluorescence turn-on effect in buffer (Figure 7b), whereas the absorption remains similar, confirming the PeT quenching mechanism of the unreacted fluorophore. Such design has been later adapted to the sensing of biothol,^45^ Peroxynitrite,^46–48^ hydrogen peroxide,^46^ and hypochlorous acid,^49^ with respective triggers connected via a self-immolative linker in a similar fashion. Notably, Guo and co-workers red-shift the wavelength of such BODIPY-type ABS sensors into the NIR by extending the conjugation through the 3- and 5-position with detailed study to inhibit the nonradiative pathways to maintain the brightness, affording **10** (FP-BDP4, Figure 7c) carrying a boronic acid for the detection of H_2_O_2_.^34^ With a peak emission at 720 nm and a ca. 30-fold turn-on, FP-BDP4 successfully detected the elevated H_2_O_2_ in an LPS-induced mouse inflammation model (Figure 7d).^35^ Another NIR example by Zhang and co-workers uses a boronic ester designed **11** (Figure 7e) to detect peroxynitrite, with a BODIPY fluorophore core with longer extended conjugation from the 3- and 5-positions, which shifts the emission maximum to 805 nm,^47^ enabling the detection of peroxynitrite in a mouse inflammation model with LPS treatment (Figure 7f). These results highlight the value of pyridium-based BODIPY probes for imaging oxidative stress in inflammatory microenvironments.

**Figure 7:**
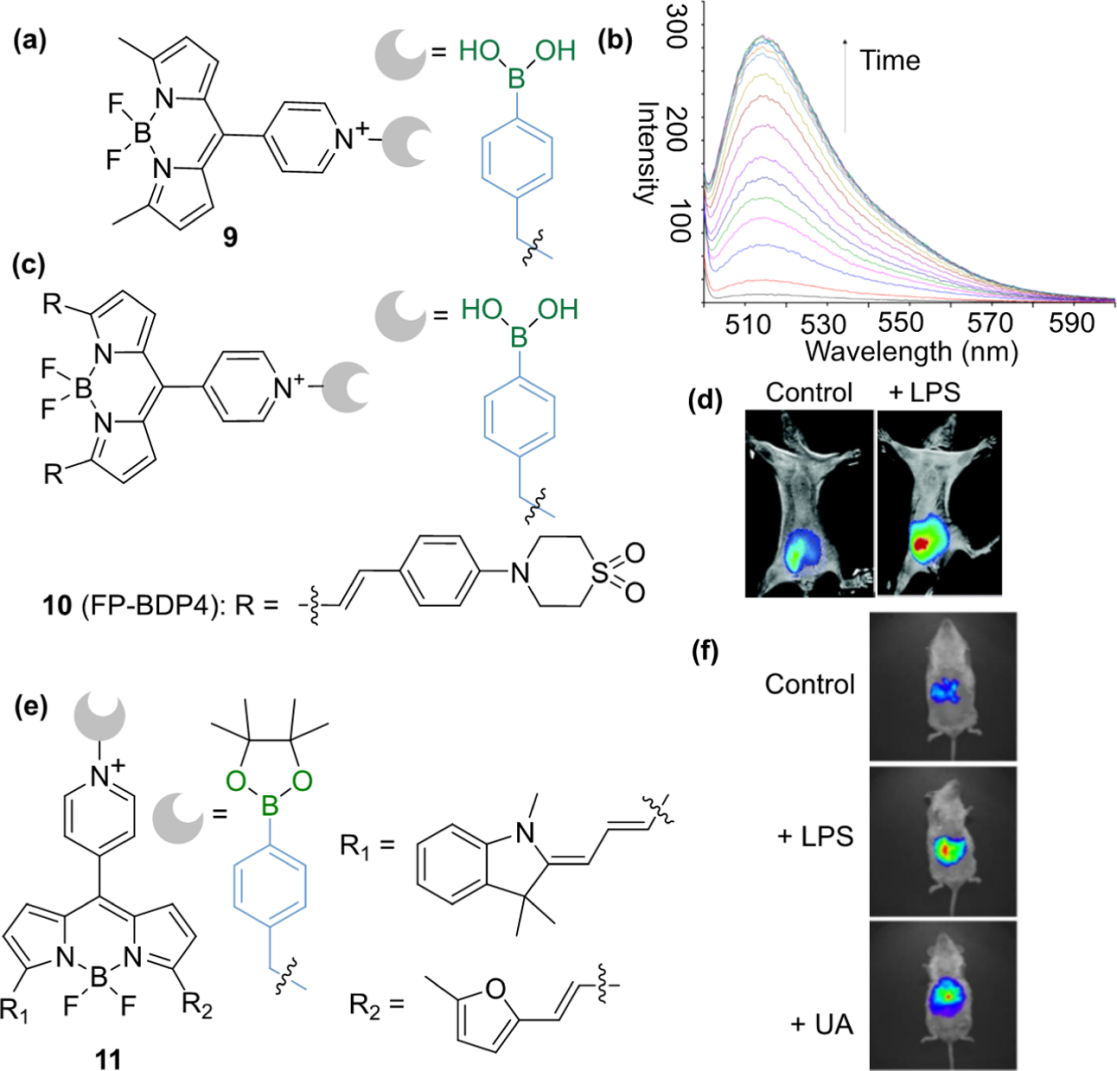
BODIPY-based ABS sensors utilizing a *meso*-pyridinium caging strategy. (a) Chemical structures of probe **9** for hypochlorite detection. (b) Fluorescence emission spectra showing concentration-dependent turn-on response. Adapted with permission from ref 33 Copyright 2017 Elsevier. (c) Chemical structure of NIR probe **10** (FP-BDP4) with extended π-conjugation at the 3- and 5-positions. (d) In vivo NIR fluorescence imaging of H_2_O_2_ in an LPS-induced mouse inflammation model using **10** (FP-BDP4): control (left) versus LPS treatment (right). Adapted with permission from ref 34 Copyright 2021 The Royal Society of Chemistry. (e) Chemical structure of an extended-conjugation BODIPY probe **11** for peroxynitrite detection with emission maximum at 805 nm. (f) In vivo NIR fluorescence imaging of peroxynitrite in a mouse inflammation model: control, Lipopolysaccharide (LPS) treatment, and Uric Acid (UA) treatment groups. Panel (f) adapted with permission from ref 34 Copyright 2026 Elsevier.

## 3 Heptamethine-Based Activatable Probes

Polymethine fluorophores represent a large class of tunable dye scaffold, which are singly charged molecules composed of two heterocycle end groups connected by an odd number of methine units (sp^2^ carbon) (Figure 8a). The absorption wavelengths are dependent on the number of the methine units, where addition of –CH=CH– predictably results in a ca. 100 nm red-shift. The identity of two heterocycles can also tune the wavelength of the fluorophore.^57^ These characters lead to the >700 nm absorption and emission of Cy7 (Figure 8b), a popular heptamethine cyanine dye for NIR imaging, which comes with two indolinium heterocycles and seven methine units. By further elongating the methine bridge^58,59,65^ and the select use of red-shifted end groups,^65,68^ polymethine dyes have been further shifted to the SWIR region as always-on probes for animal imaging with enhanced contrast.^60–62^ A summary of the probes discussed in this section is provided in Table S2.

**Figure 8:**
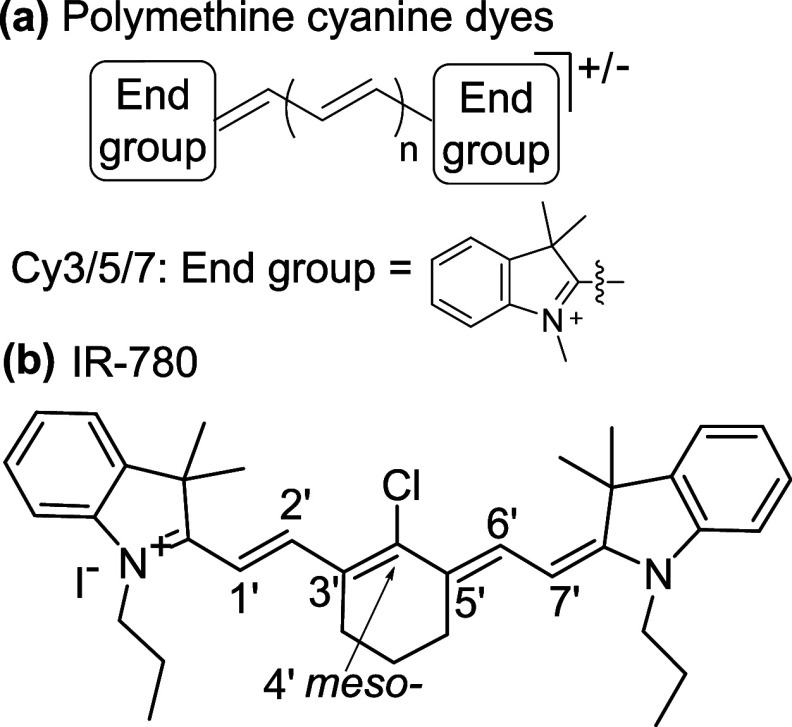
(a) The general structure of polymethine dyes. (b) The structure of IR-780, a common heptamethine fluorophore.

Despite their beneficial photophysical properties for animal imaging, pristine polymethine dyes lack a site for direct on/off modulation for integration into ABS sensors.^64^ To overcome this limitation, a series of modifications have been performed on polymethine dyes,^67^ especially heptamethine dye for ABS sensor development, which will be discussed in this section.

### 3.1 Heptamethine Dyes with Phenolate-to-Quinone Activation

The singly charged character of polymethine dyes serves as a structural foundation for the intense, red-shifted absorption of this dye family, which can be purposely reprogrammed for incorporation into ABS sensors. Toward this end, Shabat and co-workers first introduced a “donor-two-acceptor” design based on phenolic oxygen modification of NIR cyanine scaffolds with probe QCy7 (Figure 9).^68^ In this design, the free fluorophore in its ON state can access cyanine-like resonance structures (**12b**, Figure 9) that display NIR absorption and emission. With selective triggers attached to the phenolic positions via ether linkage, the dye adopts an OFF state, where the phenolic ether disrupts the electron-donating effect, resulting in a double charged structure without cyanine-like NIR emission (**12a**, Figure 9). Upon activation, the free phenolic/phenolate donor is restored, re-establishing the donor–acceptor delocalization and cyanine-like resonance character of the polymethine scaffold and turning ON fluorescence. Using this elegant strategy, the authors coupled the fluorophore to a boronic acid through a self-immolative linker, with sulfonate groups for improved water solubility, to create the cyanine-based H_2_O_2_ sensor **13** (Figure 10a).^68^ This probe showed a significant turn-on response upon detection of endogenously generated H_2_O_2_ in an LPS-treated mouse model (Figure 10b). The result demonstrates that activatable heptamethine probes can be used to visualize inflammatory oxidative stress in vivo. As follow-ups, the “donor-two-acceptor” design was further expanded by Shabat to other heptamethine dyes^69^ and the detection of other bioanalytes by using respective trigger moieties such as phenylboronate ester for H_2_O_2_, DNBS for thiols (GSH/Cys/Hcy), and β-galactose for β-galactosidase activity.^69^

**Figure 9:**
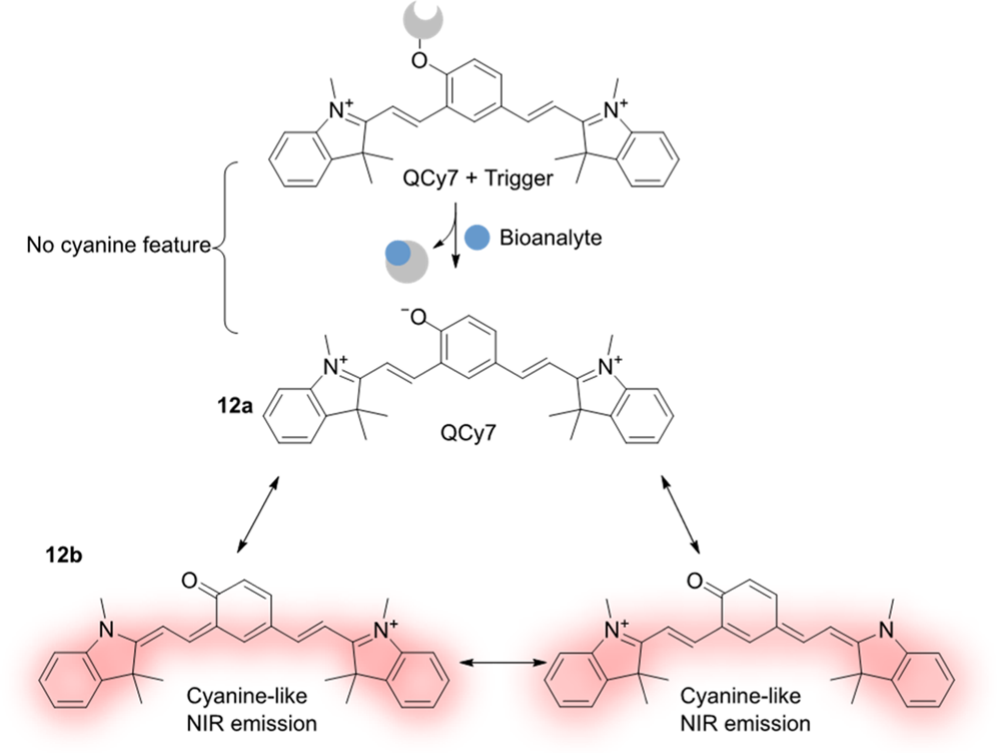
Design and turn-on mechanism of “donor-two-acceptor” QCy7-based ABS sensors.

**Figure 10:**
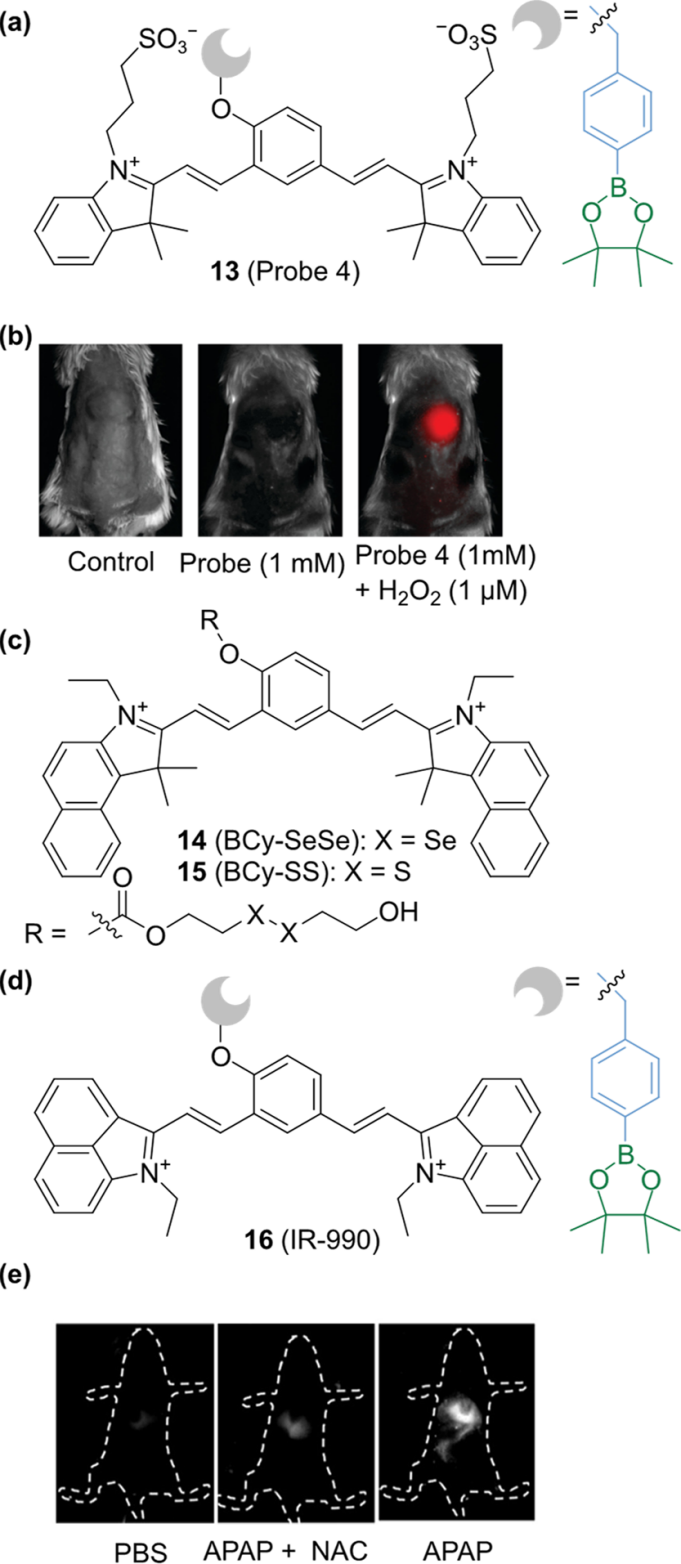
Representative “donor-two-acceptor” heptamethine dye-based ABS sensors and their in vivo applications. (a) Chemical structure of **13**. (b) In vivo NIR fluorescence imaging of endogenously generated H_2_O_2_ in an LPS-treated mouse model using **13**. Adapted with permission from ref 50 Copyright 2019 American Chemical Society. (c) GSH-responsive probes **14** and **15**. (d) Chemical structure of **16** (IR-990), a SWIR H_2_O_2_ probe incorporating benzo­[*cd*]­indolium end groups with a boronic ester trigger. (e) SWIR fluorescence imaging of H_2_O_2_ detection in mouse liver after acetaminophen stimulation using IR-990. Adapted with permission from ref 52 Copyright 2022 American Chemical Society.

Since its first report, this type of heptamethine dye-based ABS sensors has been expanded to encompass a variety of triggers and detection wavelengths. Owing to the modularity, a couple of triggers have been attached to the scaffold for in vivo sensing, including the detection of β-galactosidase activity,^69^ GSH,^51^ peroxynitrite,^70,71^ nitroreductase,^72^ and H_2_O_2_.^52^ Owing to its origin from the heptamethine dye scaffold, methodologies in tuning the wavelengths of heptamethine dyes also apply to the “donor-two-acceptor” sensors. By fusing another benzene ring on the indolinium heterocycle, Chen and co-workers shifted the emission to 728 nm in their GSH sensor **14** (BCy-SeSe) and **15** (BCy-SS, Figure 10c), which enables the visualization of GSH levels in a mouse middle cerebral artery occlusion (MCAO) model (Figure 10d).^51^ Alternatively, with two benzo­[*cd*]­indolium end groups, a heterocycle was seen in SWIR dyes FD-1080 ^66^ and IR-1048, Xiong and co-workers created a H_2_O_2_ probe **16** (IR-990), enabling SWIR detection of an enhanced H_2_O_2_ level in liver after acetaminophen stimulation (Figure 10e,f).^52^ Zhang and co-workers incorporated a chromenylium heterocycle, also seen in Chrom-series heptamethine dyes,^63^ and achieved IRBTP-B probe for peroxynitrite, with emission maximum around 950 nm.^70^

### 3.2 Miscellaneous Heptamethine Biosensors with Trigger at the *meso*-Position

The *meso*-position of heptamethine dyes represents a unique position for modification owing to their maintenance of symmetry of the fluorophore and its perturbation in the electronic structure of the fluorophore. In a recent report, Chen and co-workers developed a trigger connection scheme with pyridinium at the *meso*-site, similar to the success in the BODIPY (Section 2.2) and the xanthene dyes.^73,74^ In their design, the 4-position of pyridinium connects to the *meso*-carbon in Cy7, and the nitrogen atom connects to a trigger via a self-immolative linker (Figure 11a).^75^ In the OFF state, the cationic pyridinium serves as a strong electron-withdrawing quencher that suppresses Cy7 fluorescence via d-PeT quenching. Upon activation, analyte-triggered cleavage of the self-immolative linker relieves quenching and restores the fluorescence ON state. The authors demonstrate compatibility with the sensing of a panel of biological events, including biothiol, superoxide, nitroreductase, and esterase, all resulting in turn-on folds between 32 and 65 in in vitro assays. Besides connecting to the responsive trigger, the positive charge on pyridinium salt renders the clearance pathway to the kidney, different from the liver clearance as in most cyanine fluorophores. This enables faster clearance and lower background for imaging acute kidney injury in the mouse model upon LPS treatment with their superoxide sensing probe **17** (CyP-DPP, Figure 11b). At the same time, pyridinium was reported to serve as a trigger connection adapter for making the ABS probe, where the nitrogen atom connects to the *meso*-position of the fluorophore and the 4-site is modified with an amine attached to a boronic ester trigger for H_2_O_2_ via a self-immolative linker.^53^

**Figure 11:**
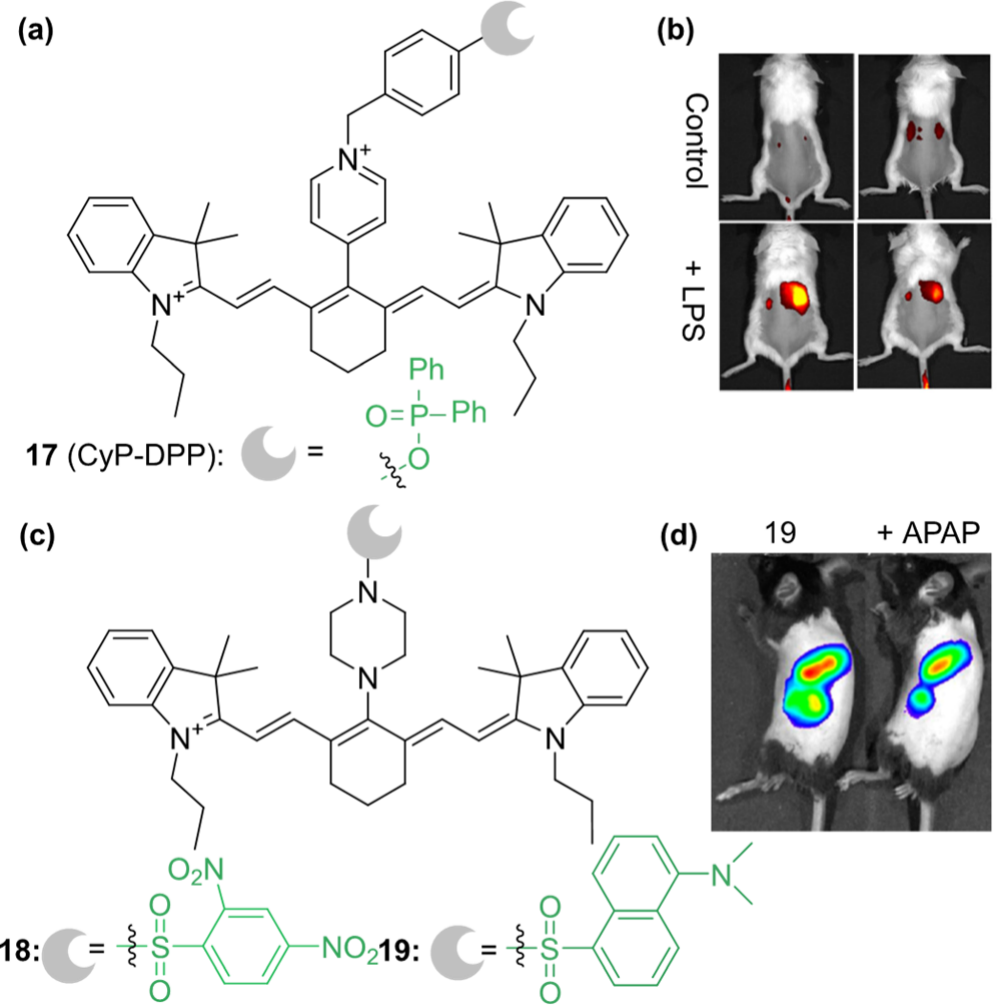
Heptamethine-based ABS sensors with *meso*-position modification. (a) Chemical structure of *meso*-pyridinium probe **17** (CyP-DPP) with a diphenylphosphinate trigger for superoxide detection. (b) In vivo NIR fluorescence imaging of acute kidney injury in control and LPS-treated mice. Adapted with permission from ref 75 Copyright 2025 American Chemical Society. (c) Chemical structures of *meso*-piperazine-linked heptamethine probes **18**, **19** with for GSH detection. (d) In vivo NIR fluorescence imaging of GSH levels in acetaminophen (APAP)-treated mice using probe **19**. Adapted with permission from ref 54 Copyright 2014 American Chemical Society.

Another method for designing heptamethine dye-based ABS sensors using its *meso*-modification is by introducing the trigger via a piperazine linkage. In 2014, Yoon and co-workers designed a selective GSH sensor by attaching a sulfonamide as a trigger for GSH (Figures 11c, 18, and 19).^54^ After trigger cleavage, one of the piperazine nitrogen becomes a secondary amine, which results in a significant fluorescence turn-on with high specificity to GSH, enabling the detection of a lowered GSH level in mouse after acetaminophen treatment (Figure 11d). This application highlights the utility of heptamethine ABS probes for monitoring thiol depletion associated with drug-induced oxidative injury in vivo. Although one later report developed a boronic ester probe for H_2_O_2_ sensing with a mild turn-on ratio,^76^ which was attributed to PeT quenching on the intact probe, most subsequent ABS sensors with this type of Cy7 connection rely on trigger moieties carrying additional quenching functionalities such as nitro groups or metal ions,^77–81,92^ which may limit the compatibility of this scaffold.^82^

Besides these turn-on ABS probes, the *meso*-position can be leveraged for the designing of ratiometric probes owing to the bis-dipolar resonance form when this site is replaced with a strong electron-donating group.^83,84^ While the Cy7 structure exhibits NIR absorption and emission with the positive charge spread out along the methine bridge, its bis-dipolar resonance form, with positive charge localized at the *meso*-position, contributes to the blue-shifted emission in the visible region (Figure 12). To harness this feature, heptamethine dyes bearing a *meso*-amino group can be used as a fluorophore with major dipolar resonance contribution owing to the positive charge stabilizing effect of the amino group and thus display blue-shifted wavelengths. Upon attachment of the trigger to the amino group, the electron-donating capacity is reduced, bringing more cyanine-like red-shifted absorption and emission in the NIR region (Figure 12). This trigger attachment scheme has been incorporated into ABS sensors for H_2_O_2_,^85^ superoxide,^86,87^ and H_2_S.^88^

**Figure 12:**
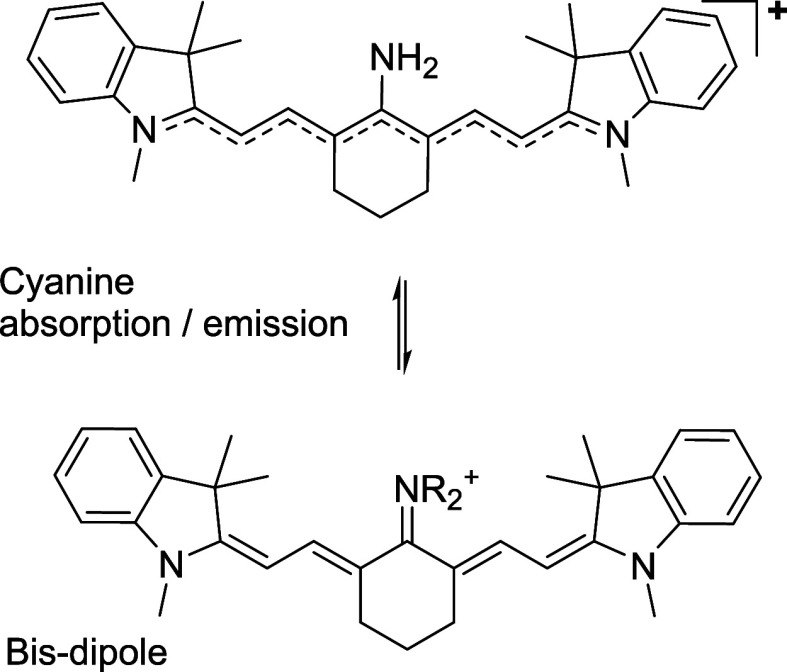
Resonance structures of *meso*-amino-substituted heptamethine dyes.

With a more sophisticated attachment scheme, Zeng and co-workers developed a γ-glutamyl transpeptidase (GTT) probe **20** (CFC-GSH, Figure 13a),^55^ utilizing the intramolecular rearrangement that results in the *meso*-substitution changed from a thio ether to an amine. The increased bis-dipole resonance contribution of the product with *meso*-amino group results in a strong blue-shift in both absorption and emission of the fluorophore, furnishing a ratiometric GTT probe for metastatic tumor detection (Figure 13c). Overall, the ratiometric property of these kinds of probe is beneficial for simple imaging experiments to the intrinsic control; however, in animal imaging, their far-red fluorescence may exhibit shallower penetration depth compared to the NIR emission, potentially compromising the detection of in vivo biological processes. To overcome this issue while taking advantage of the two resonance forms, Fan and co-workers leverage a intramolecular S-to-N rearrangement during trigger cleavage to furnish a NIR turn-on probe **21** (Tg-PEG) for GSH (Figure 13c).^56^ This design conjugates the *meso*-amino group of the Cy7 fluorophore with an RGD peptide for cancer targeting via an appropriately separated disulfide trigger. Upon reduction by GSH, the free thiol replaces the *meso*-nitrogen, resulting in less contribution of the bis-dipolar resonance form and a NIR turn-on response from the cyanine form. This probe has been successfully applied in the visualization of liver metastases in a mouse model (Figure 13d), which has potential clinical implications for early detection of occult metastatic lesions and improved intraoperative guidance in hepatobiliary oncology.

**Figure 13:**
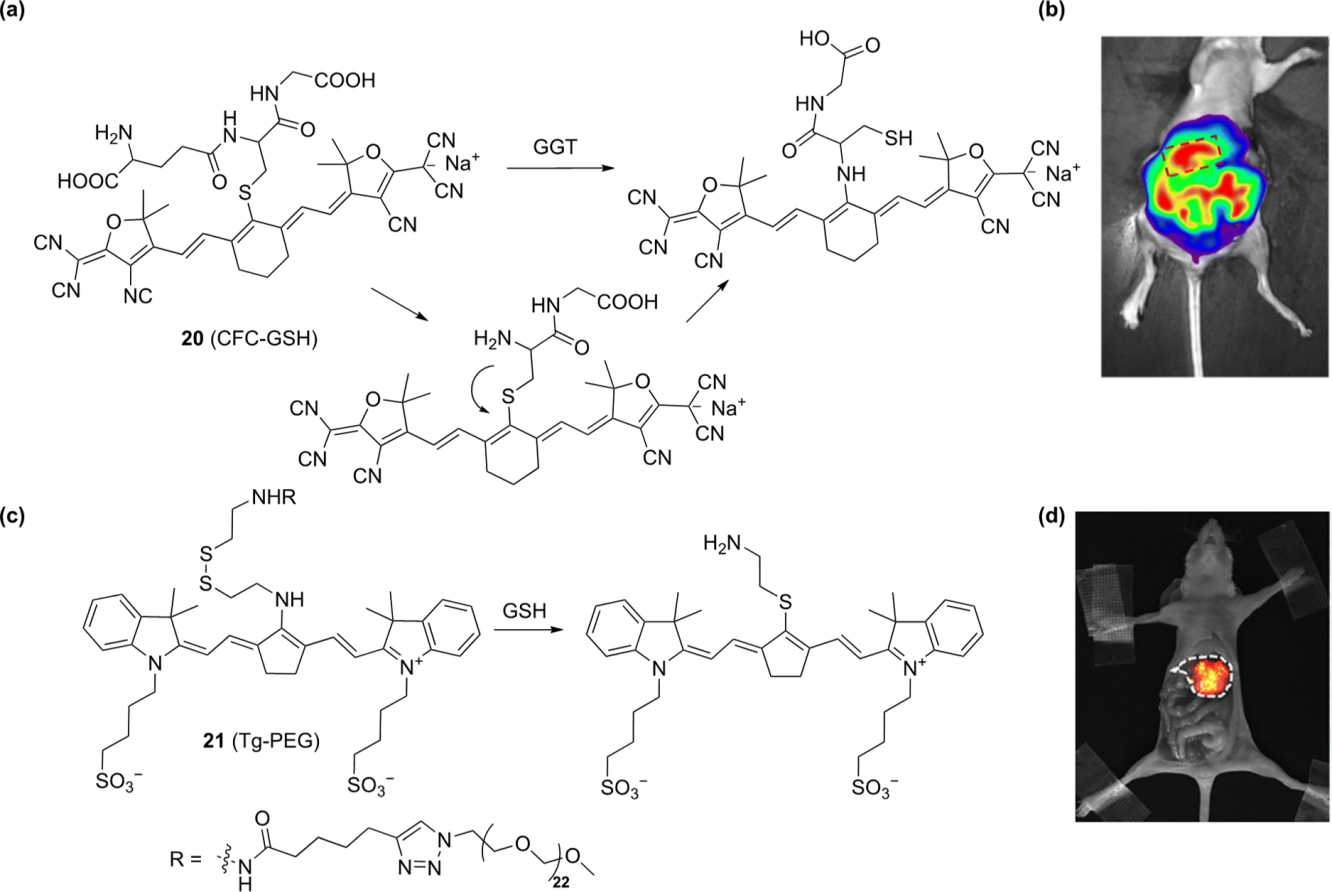
Heptamethine-based ABS sensors utilizing intramolecular rearrangement at the *meso*-position. (a) Chemical structure of **20** (CFC-GSH) and its GGT-triggered activation mechanism. (b) In vivo fluorescence imaging of metastatic tumors using CFC-GSH. Adapted with permission from ref 55 Copyright 2025 Royal Society of Chemistry. (c) Chemical structure of **21** (Tg-PEG) and its GSH-triggered activation mechanism. (d) In vivo NIR fluorescence imaging of liver metastases using Tg-PEG. Adapted with permission from ref 56 Copyright 2025 Wiley.

## 4 Hemicyanine-Based Activatable Fluorescent Probes

Unlike polymethine cyanine dyes, where the heteroatoms are embedded within a heterocyclic ring and less accessible for fluorescence modulation, hemicyanine dyes replace one side of the heterocycle with a free hydroxyl or amino handle, providing it a handy site for attaching triggers (Figure 14). The facile conversion of Cy7 fluorophores to NIR hemicyanine dyes further makes this scaffold attractive for the construction of ABS sensors for in vivo detection. In the OFF state, masking the phenolic hydroxyl or amino group attenuates its electron-donating ability and suppresses intramolecular charge transfer (ICT) to the indolium acceptor, resulting in a diminished NIR fluorescence. The phenolic hydroxyl group on hemicyanine is a strong electron-donating group when it is deprotonated in physiological buffer. Upon reaction with the target bioanalyte, trigger cleavage regenerates the free hydroxyl or amino group, restores donor strength and ICT, and thereby produces the fluorescent ON state (Figure 14). The straightforward trigger attachment profile, the NIR absorption and emission properties, and the rich modification strategies on hemicyanine dyes make it a popular scaffold for making NIR to SWIR ABS sensors. A summary of the probes discussed in this section is provided in Table S3.

**Figure 14:**
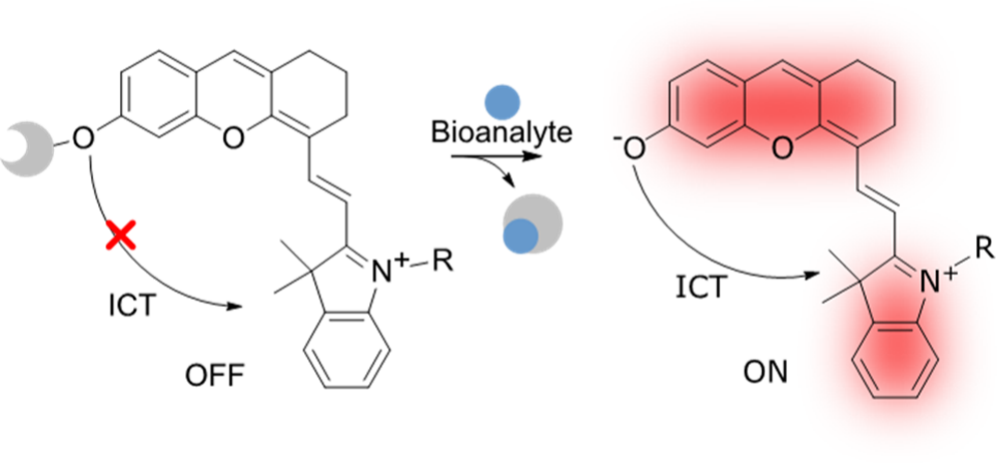
Turn-on mechanism of hemicyanine-based ABS sensors.

The concept of ABS sensors based on hemicyanine was first established by Lin and co-workers in 2012.^89^ In their seminal report, they constructed probe **22** (NIR-H_2_O_2_) by connecting a Boronic acid to the hemicyanine scaffold via a self-immolative linker (Figure 15a). Upon oxidative cleavage of the boronate, the resulting phenol intermediate initiates spontaneous 1,6-elimination, fragmenting the linker and releasing the free hemicyanine fluorophore with emission at 708 nm and a 180-fold turn-on (Figure 15b). The significant fluorescence turn-on response led to the sensitive detection of an endogenous elevated level of H_2_O_2_ in the mouse model with LPS-mediated inflammatory response (Figure 15c). Following similar O-site conjugation principles with direct boronate attachment, Zhang,^107^ Peng,^108^ and Zhang^109^ developed similar probes where oxidative cleavage converts the phenylboronate to a phenolic group, thereby constructing the ICT system and turning on fluorescence. Additionally, in Lin’s first report, the hemicyanine scaffold was also directly attached via a sulfate ester to 2,4-dinitrobenzenesulfonate for thiol detection,^89^ allowing for the first NIR imaging of endogenous thiols in live mice. These NIR turn-on probes demonstrate how the modular hydroxyl-trigger design can be applied to address biological questions such as real-time visualization of endogenous species (e.g., H_2_O_2_ and thiols) in living mice while establishing a generalizable strategy for constructing new NIR sensors suitable for in vivo imaging of diverse disease-relevant biochemical processes.

**Figure 15:**
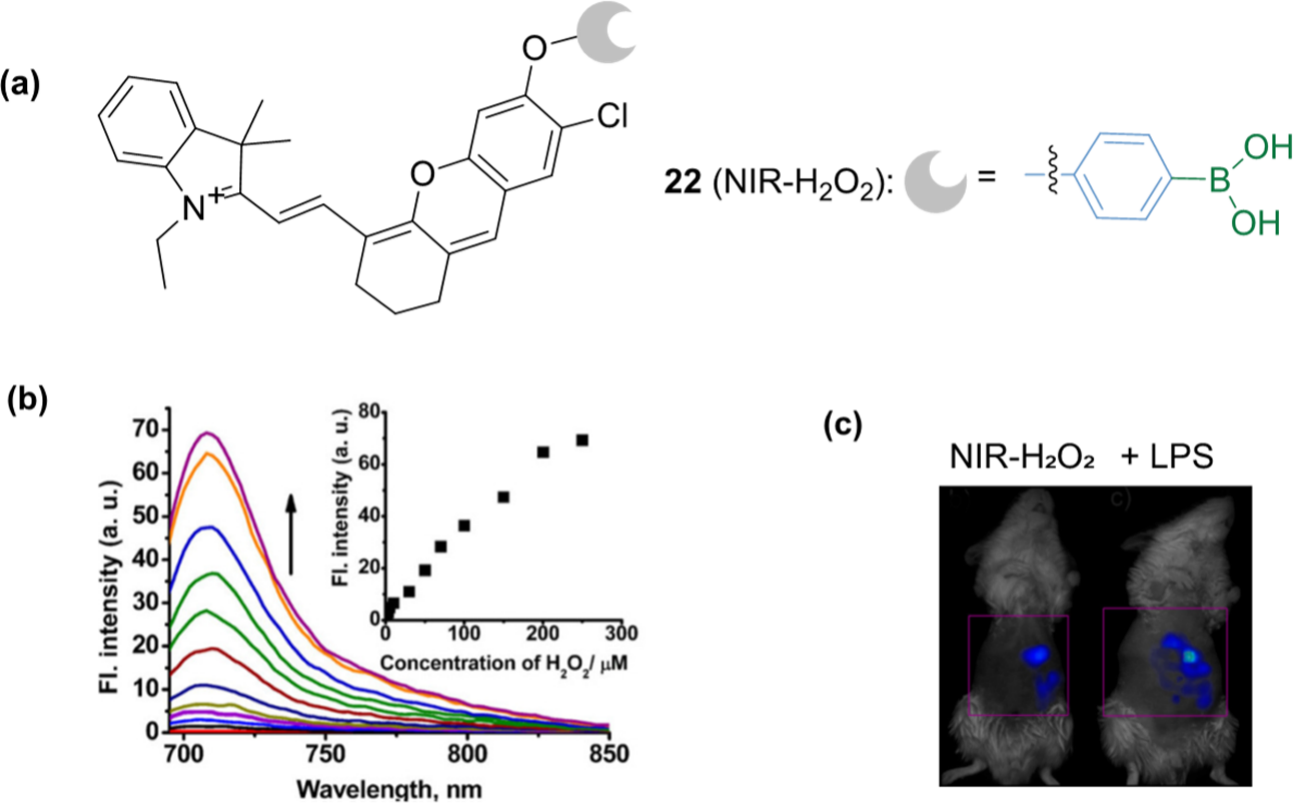
Hemicyanine-based ABS sensors for small-molecule detection. (a) Chemical structure of **22** (NIR-H_2_O_2_). (b) Fluorescence emission spectra showing concentration-dependent turn-on response upon H_2_O_2_ addition. (c) In vivo NIR fluorescence imaging of endogenous H_2_O_2_ in mice. Adapted with permission from ref 89 Copyright 2012 American Chemical Society.

Since its first design, the two main design prototypes for hemicyanine-based sensors, using either a direct connection or a self-immolative linker to a trigger, have been widely explored for NIR absorbance detection. For direct connection, a series of triggers with sulfonyl ester linkage was coupled to the hemicyanine scaffold while preserving identical activation mechanisms. Yu,^86^ Wu,^110^ and Fan^90^ developed probes by connecting PFBS groups to the phenolic oxygen for the detection of H_2_O_2_, for example, in probe **23** (Figure 16a), which enables the visualization of H_2_O_2_ in a nonalcoholic fatty liver disease (NAFLD) mouse model (Figure 16b).^90^ Yuan and co-workers introduced DNBS as a responsive moiety connected to the phenolic oxygen through sulfonyl ester linkage, creating NIR probe **24** (HXPIS, Figure 16c).^91^ Li and co-workers employed the same DNBS ester design, with expansion of the conjugation structure, achieving extended absorption/emission wavelengths with enhanced photoacoustic and fluorescence signals.^111^ From a biological standpoint, sulfonate-triggered ABS probes provide a practical framework for investigating disease-associated redox processes in living systems. Specific questions that can be addressed include how mitochondrial H_2_O_2_ levels evolve during inflammatory or metabolic injury, whether elevated glutathione concentrations within the tumor microenvironment can be utilized to distinguish malignant from normal tissues, and how dynamic redox changes influence cellular stress responses or therapeutic efficacy. Thiocarbamate linkages have also been attached to construct ABS sensors for hypochlorite sensing, where Lin^92^ and Liu^93^ created probes by connecting *N*,*N*-dimethylthiocarbamate moieties to the phenolic oxygen, enabling the detection of hypochlorite in a mouse model. These examples include probes **25** (CyClOP, Figure 16d) and **26** (NFL-S, Figure 16f), both enabling the detection of an elevated level of hypochlorite in mouse inflammation models (Figure 16e,g). Other responsive triggers have also been directly connected to the hydroxyl groups on hemicyanine dyes for making ABS sensors, such as trifluoromethyl ketone for peroxynitrite detection^112^ and diphenylphosphite for H_2_S sensing.^113,114^

**Figure 16:**
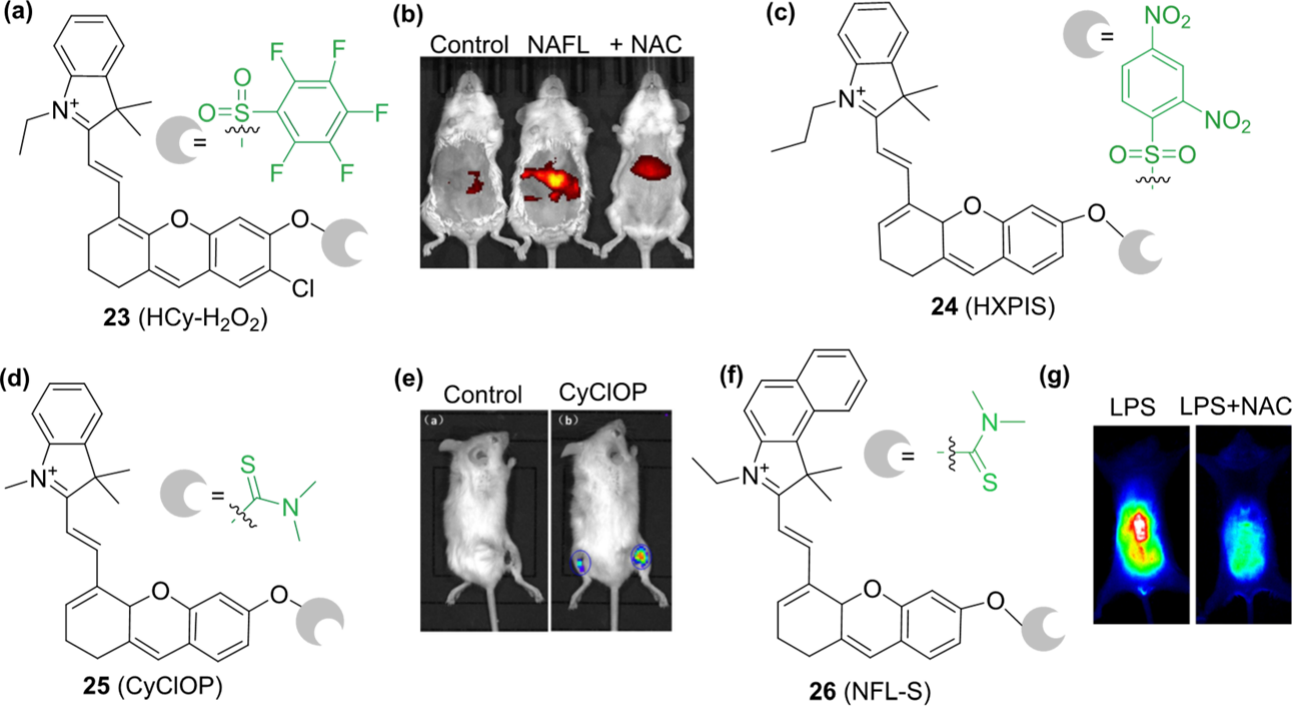
Hemicyanine-based ABS sensors with direct trigger connection. (a) Chemical structure of **23** (HCy-H_2_O_2_) with PFBS trigger for H_2_O_2_ detection. (b) In vivo NIR fluorescence imaging of an elevated H_2_O_2_ level in the NAFL mouse model and a reduced level with *N*-acetylcysteine (NAC) treatment. Reproduced with permission from ref 90 Copyright 2024 American Chemical Society. (c) Chemical structure of **24** (HXPIS) with a DNBS trigger for thiol detection. (d) Chemical structure of **25** (CyClOP) with an *N,N*-dimethylthiocarbamate trigger for HOCl detection. (e) In vivo NIR fluorescence imaging of HOCl in a mouse muscle injury model, with the right leg injured and the left leg as the control. Reproduced with permission from ref 92 Copyright 2021 Elsevier. (f) Chemical structure of **26** (NFL-S) with a thiocarbamate trigger for HOCl detection. (g) In vivo NIR fluorescence imaging of HOCl in LPS-treated mice. Reproduced with permission from ref 93 Copyright 2021 Elsevier.

On the other hand, trigger connection to hemicyanine dyes via the self-immolative linker allows more flexibility as the elimination process allows the different functional groups between the phenolic hydroxyl on hemicyanine dyes and the trigger moiety. Using this scaffold, hemicyanine dyes have been coupled to aryl azide, which release an amino group upon H_2_S detection. Yoon and co-workers designed **27** (NIR-Az) by incorporating a self-immolative benzyl linker between the phenolic oxygen and an azide trigger for H_2_S detection (Figure 17a).^94^ Upon reduction of the azide to amine, the electron-donating group triggers spontaneous 1,6-elimination to release the free hemicyanine fluorophore with over 200-fold fluorescence enhancement, enabling its detection of exogeneous H_2_S in mouse imaging (Figure 17b). Zhang and co-workers introduced an acrylate group through ester linkage at the phenolic oxygen, developing **28** (CyA) for biothiol detection (Figure 17c).^95^ Upon thiol-promoted Michael addition, followed by intramolecular cyclization, the ester bond is cleaved to release the native fluorophore, enabling the visualization of thiols in cell models (Figure 17d). This acrylate-based platform has since been broadly adopted with variations in hemicyanine core structures to improve water solubility and extend emission wavelengths.^115–118^

**Figure 17:**
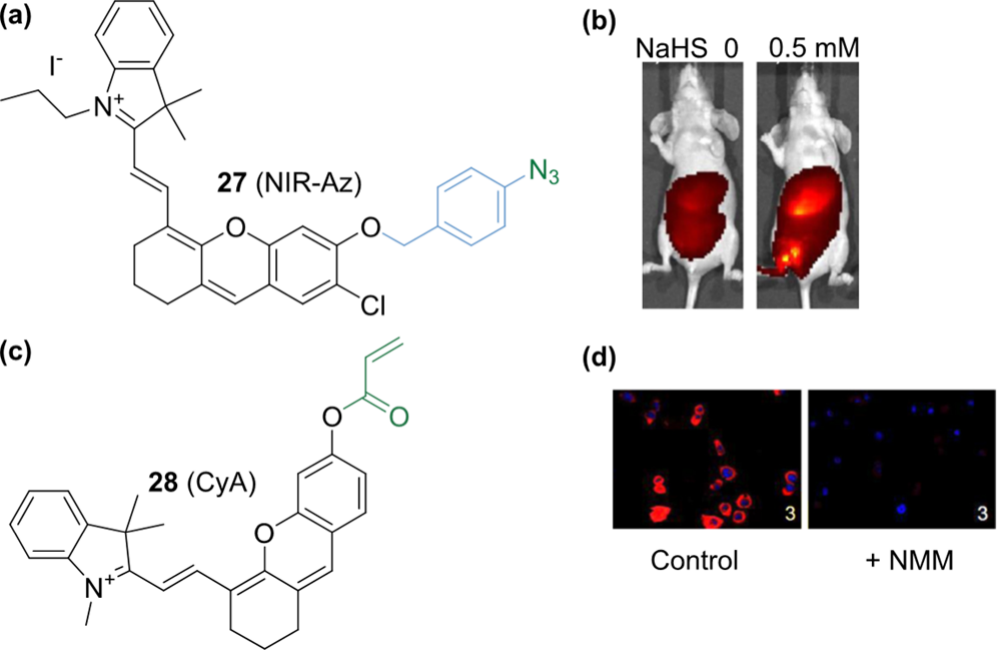
Hemicyanine-based ABS sensors with trigger attachment via self-immolative linkers. (a) Chemical structure of **27** (NIR-Az) with an azide trigger for H_2_S detection. (b) In vivo NIR fluorescence imaging of H_2_S in mice with exogenously introduced NaHS. Reproduced with permission from ref 94 Copyright 2017 Elsevier. (c) Chemical structure of **28** (CyA) with an acrylate trigger for biothiol detection. (d) Confocal fluorescence imaging of biothiols using CyA in cells with or without *N*-Methylmaleimide (NMM, a thiol scavenger). Reproduced with permission from ref 95 Copyright 2015 American Chemical Society.

Beyond sensing of small-molecule analytes, hemicyanine dyes have also been harnessed for the detection of enzyme activities by using respective substrates as a trigger moiety, which is linked to the fluorophore with self-immolative linkers when necessary. For example, Pu and co-workers designed **29** (CyGal-P), a β-galactosidase activated theranostic probe for tumor, consisting of d-galactose-caged NIR hemicyanine dye linked to a long PEG chain at the *N*-site through click reaction for improved biocompatibility (Figure 18a).^96^ Besides fluorescence turn-on in tumor-bearing mouse models, the catalyzed hydrolysis of glycoside results in a red-shift to 688 nm and enhanced absorption, which is leveraged for up to 48 °C elevation of temperature under 680 nm illumination as photothermal therapy (PTT) for cancer (Figure 18b). Ye, Chen, and co-workers developed **30** (GANP) as a NIR sensor for γ-glutamyl transpeptidase (GGT) by linking the enzyme substrate γ-Glu to the NIR hemicyanine fluorophore through a self-immolative linker *p*-aminobenzyl alcohol (Figure 18c).^97^ In this design, γ-Glu is connected to the fluorophore with an self-immolative *p*-aminobenzyl linker alcohol. GANP was stable under physiological conditions but efficiently activated upon enzyme-catalyzed cleavage to generate approximately 100-fold enhanced fluorescence (detection limit of approximately 3.6 mU/L), enabling the visualization of tumor in a mouse xenograft model, where there is overexpressed GGT activity (Figure 18d). This sensor was coupled with the RGD peptide as a cancer targeting ligand, further promoting the contrast in tumor detection.^119^ Besides the more rigid *p*-aminobenzyl alcohol linkage, more flexible linkers can also be leveraged for connection to the enzyme substrates as triggers to separate the trigger and fluorophore for better interaction with the enzyme. Toward this end, Ma and co-workers developed **31** (CYLP) as a sensor for pantetheinase activity by inserting a flexible self-immolative linker between the hemicyanine dye and the pantothenic acid substrate as a trigger; without this linkage, the probe will not responsive to the enzyme activity, highlighting the spatial requirements for detecting enzymes (Figure 18e).^98^ CYLP exhibited excellent selectivity and sensitivity with NIR emission wavelength and detected the inhibition of pentetheinase by a molecular inhibitor (Figure 18f). Beyond small-molecule detection, hemicyanine scaffolds have also been engineered for enzyme activity sensing with enhanced specificity. In a more recent report, Hu, Pu, and co-workers integrated an AND logic gate directly into the hemicyanine backbone probe for the detection of Caspase-1 activity and NO (Probe **32**, Figure 18g).^99^ Besides attaching the Caspase-1 responsive trigger via a self-immolative linker to the phenolic hydroxyl group, the authors came up with *o*-phenylenediamine modification on the indolinium heterocycle as an additional responsive trigger for NO detection. This dual-locked probe enables the specific recognition of M1 macrophages where these two biomarkers exist and was successfully applied in the visualization of M1 macrophage polarization during influenza infection and antivirus treatment (Figure 18h). Beyond these examples, a variety of enzyme substrates have been used as triggers and coupled with hemicyanine dyes for the development of respective triggers for in vivo detection of their activity, including γ-glutamyl transpeptidase^120^ and β-galactosidase.^121,122^ Such probes enable functional interrogation of enzyme activity linked to specific cancers, including ovarian, liver, cervical, and breast cancers, as well as processes such as tumor progression and cellular senescence.

**Figure 18:**
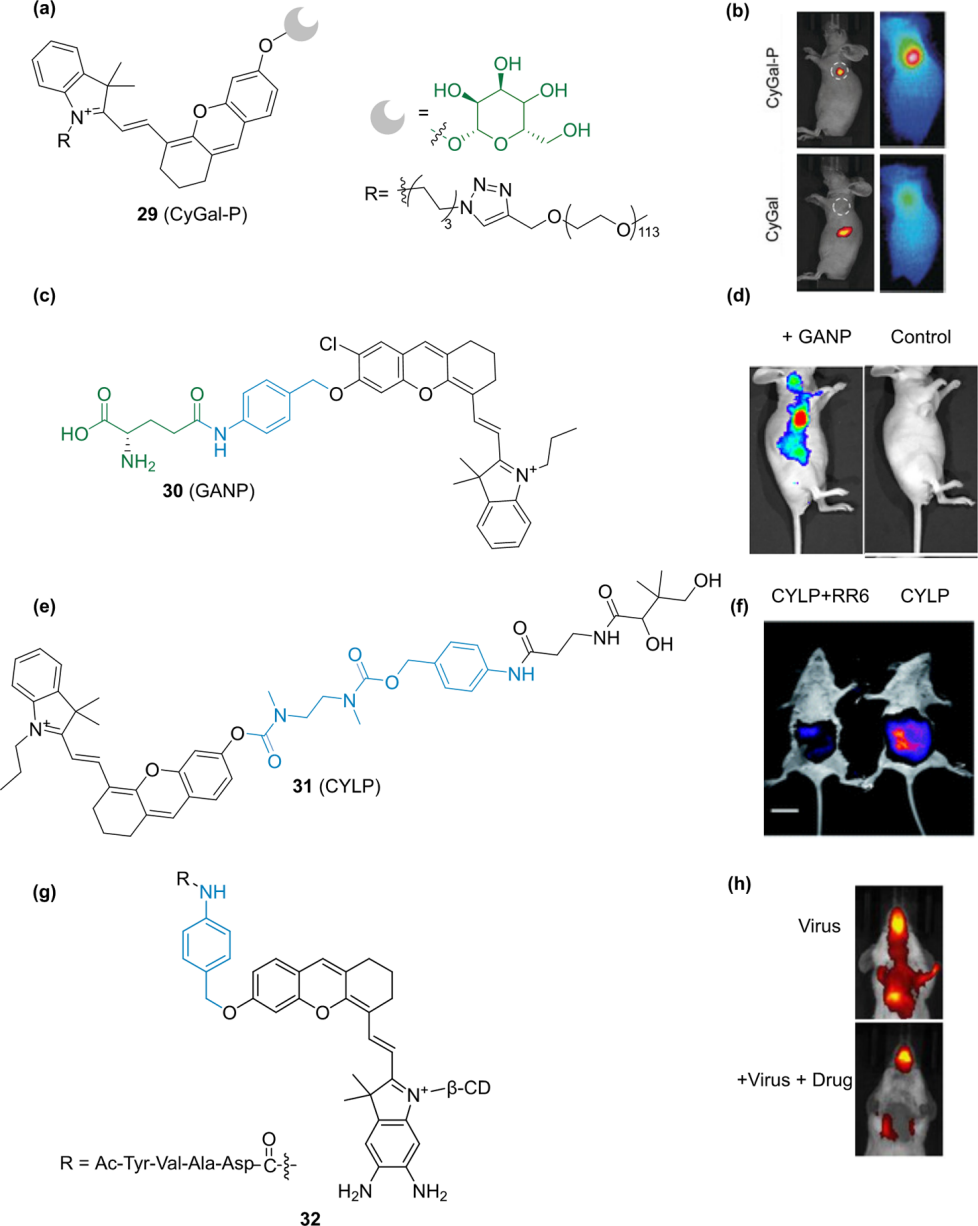
Hemicyanine-based ABS sensors for enzyme activity detection. (a) Chemical structure of **29** (CyGal-P) for β-galactosidase detection. (b) In vivo NIR fluorescence imaging of tumor by its β-galactosidase activity using CyGal-P. Reproduced with permission from ref 92. Copyright 2018 Wiley. (c) Chemical structure of **30** (GANP) with a γ-Glu trigger for GGT detection; the RGD peptide is incorporated for tumor targeting. (d) In vivo NIR fluorescence imaging of GGT activity in tumor-bearing mice at 0 min and 2 h postinjection. Reproduced with permission from ref 93. Copyright 2017 Wiley. (e) Chemical structure of **31** (CYLP) with a pantothenic acid trigger connected via a flexible self-immolative linker for pantetheinase detection. (f) In vivo NIR fluorescence imaging of pantetheinase activity in mice. Reproduced with permission from ref 93. Copyright 2020 The Royal Society of Chemistry. (g) Chemical structure of **32**, an AND logic gate probe with a Caspase-1-responsive peptide (YVAD-Ac) and an *o*-phenylenediamine trigger for NO detection. (h) In vivo NIR fluorescence imaging of M1 macrophage polarization during influenza virus infection and antiviral drug treatment. Adapted with permission from ref 99 Copyright 2025 American Chemical Society.

The structural similarity of the hemicyanine series of dyes with xanthene and polymethine fluorophores makes it convenient to further red-shift the working wavelengths with building blocks borrowed from other dye scaffolds. Liu and co-workers created **33** (HCy-HSP, Figure 19a) by substituting the endocyclic oxygen atom with a sulfur atom in the linkage between hemicyanine and the responsive moiety, which red-shifts the absorption to 720 nm and emission to 787 nm. This fluorophore was then coupled with a 2,4-dinitrophenyl trigger for the detection of H_2_S and used to detect an elevated H_2_S level in a mouse inflammation model (Figure 19b).^100^ Yuan and co-workers replaced the indolium heterocycle in hemicyanine dye with a pyrylium moiety probe **34** and **35** (Figure 19c),^101^ which is being extensively explored in polymethine field.^123^ While red-shifting the absorption wavelength to >800 nm and emission maximum around 900 nm, the replacement with the pyrylium moiety also decreases the acidity, making the free fluorophore less fluorescent at physiological pH. The authors then introduced chloro substitution at the *ortho*-position of the hydroxyl group, decreasing the p*K*_a_ from 7.8 to 6.5, rendering it useful in an animal context. The team then demonstrated its modularity by constructing three probes through strategic O-site functionalization with different responsive moieties: aryl boronate ester for peroxynitrite detection (**34**), disulfide bond for GSH detection (**35**), and phosphate ester for ALP detection (**36**, Figure 19c). All three probes successfully detected their substrates in vivo in SWIR imaging; for example, in the detection of an increased peroxynitrite level and the decreased GSH level in liver injury by APAP treatment (Figure 19d). Following these designs, Huang and co-workers extended the conjugation system on both ends and the polyene linkage of the hemicyanine scaffold and achieved emission at 1088 nm with their HBC4 fluorophore, which was coupled with a thiocarbamate trigger and inflammation-targeted tripeptide for detection of hypochlorite (**37**, Figure 19e) and applied it for imaging of colitis in a mouse model (Figure 19f).^102^

**Figure 19:**
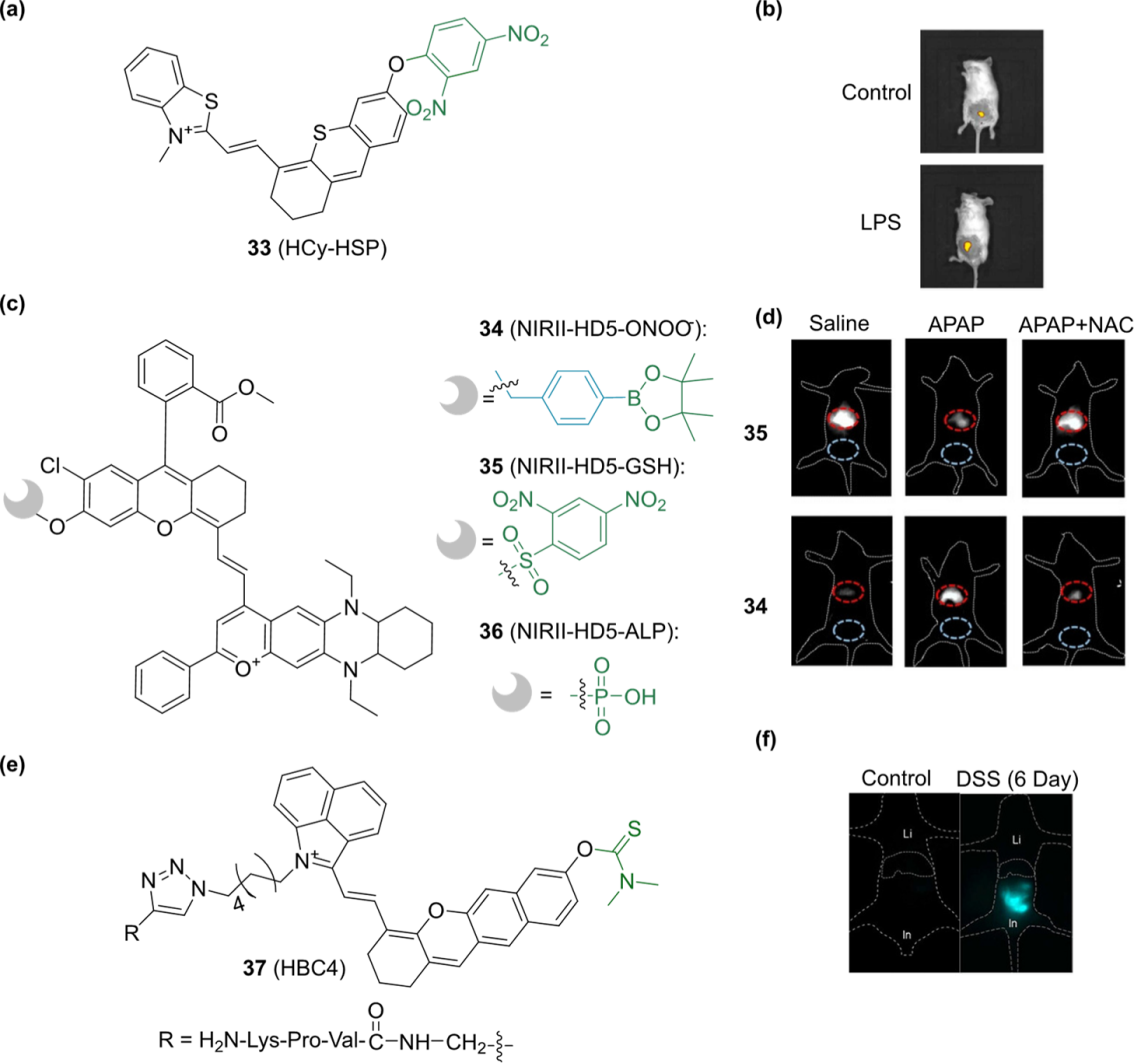
Red-shifted hemicyanine-based ABS sensors for NIR-II/SWIR imaging. (a) Chemical structure of **33** (HCy-HSP) with a 2,4-dinitrophenyl trigger for H_2_S detection. (b) In vivo NIR fluorescence imaging of H_2_S in control and LPS-treated mice. Adapted with permission from ref 100 Copyright 2021 Elsevier. (c) Chemical structures of pyrylium-based hemicyanine probes: **34** (NIRII-HD5-ONOO^–^), **35** (NIRII-HD5-GSH), and **36** (NIRII-HD5-ALP). (d) In vivo SWIR imaging of peroxynitrite and GSH in liver using **34** and **35** in mouse treated with APAP, with or without *N*-acetyl cysteine (NAC), a hepatoprotective medicine. Adapted with permission from ref 123 Copyright 2022 Wiley. (e) Chemical structure of **37** (AIR) for hypochlorite detection. (f) In vivo SWIR imaging of mouse colitis induced by DSS (dextran sulfate sodium) treatment with oral administration. Adapted with permission from ref 102 Copyright 2024 American Chemical Society.

Further pushing the wavelength of hemicyanine fluorophores into the SWIR region, Pu and co-workers applied the extension of the methine bridge in hemicyanine dyes to achieve longer emission wavelengths.^103^ In this report, the authors leveraged aldehyde homologation reaction to systematically create a palette of hemicyanine fluorophores, with varying methine bridge length. Similar to the polymethine dyes, the addition of two CH units results in a predictable ∼100 nm red-shift, yielding HCS6 with emission beyond 1000 nm, which contains six CH units and sulfur substitution of endocyclic oxygen (Figure 20a). The authors then incorporated a PEG chain for improved biocompatibility and coupled the HCS6 fluorophore with cathepsin B (Casp B)-responsive peptide sequence, acetyl-valine-citrulline, via a self-immolative linker to construct SWMP for activatable biosensing of Casp B activity as a tumor biomarker (**38**, SWIMP, Figure 20b). This probe successfully detected lung metastasis in a 4T1 tumor-bearing mouse model using SWIR imaging (Figure 20c).

**Figure 20:**
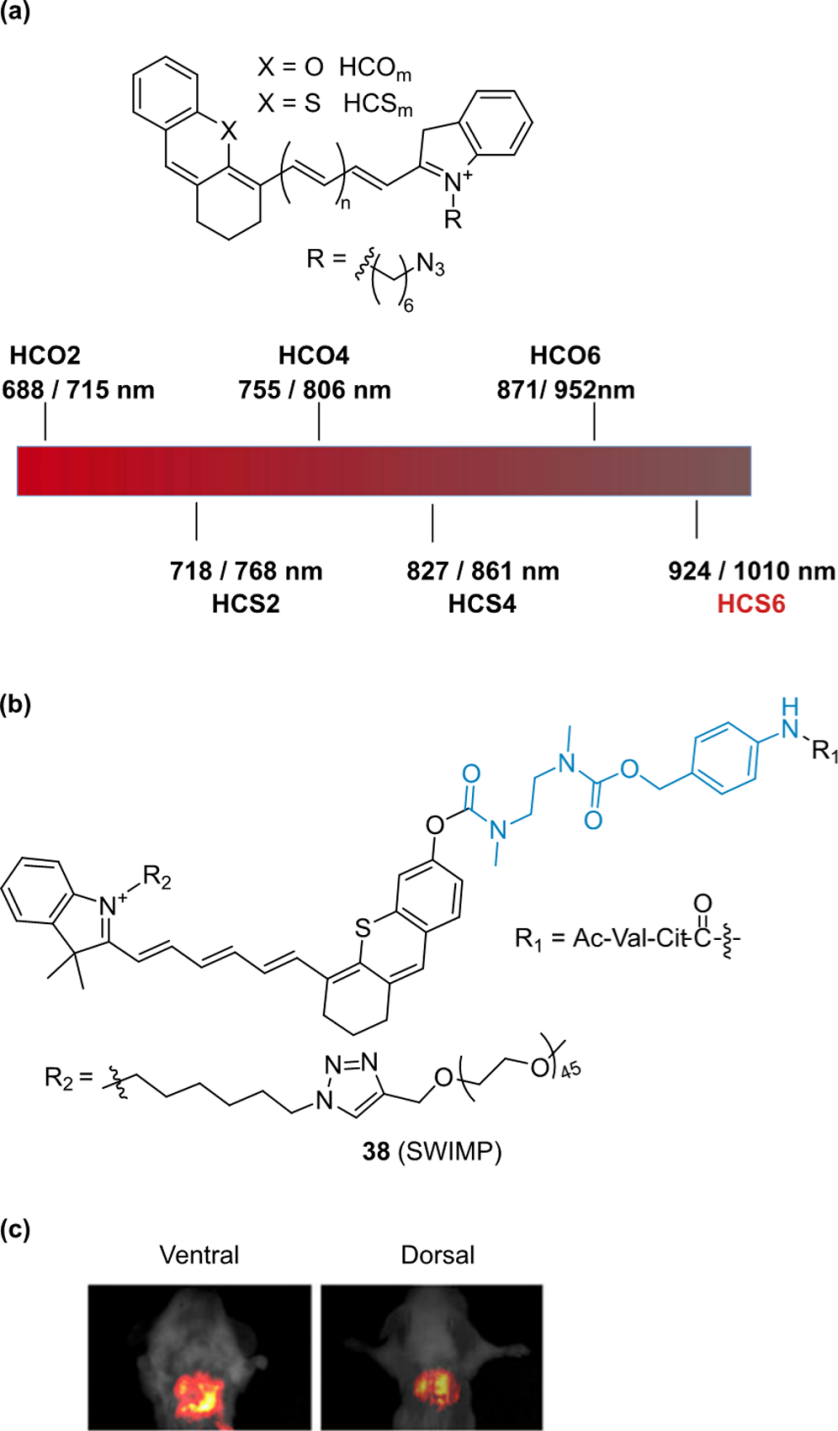
Red-shifting hemicyanine dyes by methine bridge extension. (a) Tunable emission wavelengths of hemicyanine scaffolds: HCO series (X = O) and HCS series (X = S) with varying methine bridge lengths. (b) Chemical structure of **38** (SWIMP) with the cathepsin B-responsive peptide (Cit-Val-Ac) and PEG chain for improved biocompatibility. (c) In vivo SWIR fluorescence imaging of lung metastasis in 4T1 tumor-bearing mice: ventral (left) and dorsal (right) views. Adapted with permission from ref 103 Copyright 2025 American Chemical Society.

Moving further from modifying building blocks of hemicyanine dyes with knowledge from other fluorophore scaffolds, the boundary of the hemicyanine scaffold has been expanded to allow for merging of hybrid fluorophores to construct hemicyanine dye-like fluorophores for ABS sensors. In this regard, Lei and co-workers integrated the structural features of rhodamine 6G and polymethine dyes, furnishing **39** (Rap/Py-2) fluorophores as SWIR probes for ratiometric ABS sensors (Figure 21a). In Rap, caging the secondary amine of Py-2 as a carbamate attenuates its electron-donating character, leading to blue-shifted absorption and emission relative to those of the free amine form. Upon analyte-triggered cleavage, the free amine is regenerated, restoring the original electronic structure and enabling the ratiometric ON response, making it a handy scaffold for designing ratiometric sensors.^104^ By incorporating a nitroreductase substrate as the trigger in **40a** (Rap-N), the authors were able to ratiometrically visualize the activity of nitroreductase on 4T1 tumors in live mice with or without a nitroreductase inhibitor (Figure 21c). In another study, Guo and co-workers used a flavylium end group in place of indolinium^124^ and later with a BODIPY fluorophore in **41** (BC–OH, Figure 21d),^105^ furnishing SWIR scaffolds for ABS sensor incorporation. In BC–OH-based probes, masking the phenolic donor shifts the fluorophore into a weakly emissive OFF state, whereas trigger cleavage restores the free donor and recovers the long-wavelength-absorbing/emissive ON state. Notably, probes based on BC–OH exhibit a well-separated absorption profile before and after probe activation (Figure 21e), ensuring the selective excitation of the free BC–OH absorption, which provides minimal background. This feature makes their cysteine sensor **42** (BC-Cys) with an acrylate trigger exhibit a 65-fold turn-on in vitro conditions; their boronic ester probe **43** (BC–H_2_O_2_) also shows a large fluorescence enhancement in an acetaminophen-triggered liver inflammation model (Figure 21f).

**Figure 21:**
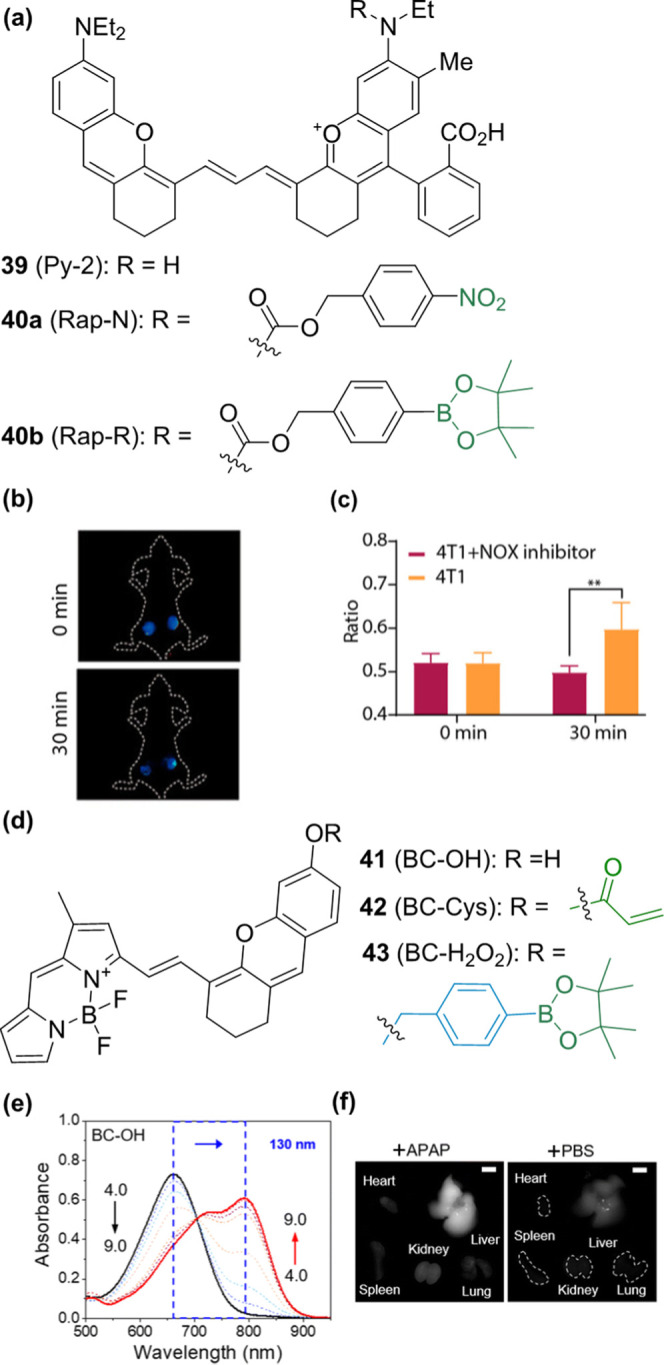
Hybrid fluorophore scaffolds for NIR/SWIR ABS sensors. (a) Chemical structures of the Py-2 fluorophore and Rap series ABS sensors. (b) In vivo SWIR fluorescence imaging of nitroreductase activity with Rap-N in 4T1 tumor-bearing mice at 0 and 30 min postinjection. (c) Ratiometric quantification of nitroreductase activity without (right) and with (left) nitroreductase inhibitor dicoumarol (200 μM). Adapted with permission from ref 104 Copyright 2022 American Chemical Society. (d) Chemical structures of the BODIPY-chromene hybrid scaffold. (e) UV–vis absorption spectra of BC–OH showing 130 nm spectral shift between pH 4.0 and 9.0. (f) Ex vivo organ imaging comparing APAP-induced liver injury versus PBS control. Adapted with permission from ref 105 Copyright 2023 Royal Society of Chemistry.

On a separate track, Zhang and co-workers synthesized a panel of merocyanine dyes **44–46** (Chrodol-1–3, Figure 22a), with similar hydroxyl handle for trigger attachment but varying fluoro-substitution to lower the p*K*_a_.^106^ Among these dyes, Chrodol-3 with two chloro-substitutions exhibits the lowest p*K*_a_ at 6.05, ensuring sufficient deprotonated population that is bright under physiological conditions. The authors then installed a boronic ester trigger to mask the phenolic hydroxyl group on Chlorodol-3 to make **47** (PN-910, Figure 22b). While showing selectivity upon detection of H_2_O_2_ with ROS, the deprotonated state requirement for the free fluorophore makes it useful for detection of ROS under alkaline conditions, such as cystitis and colitis (Figure 22c).

**Figure 22:**
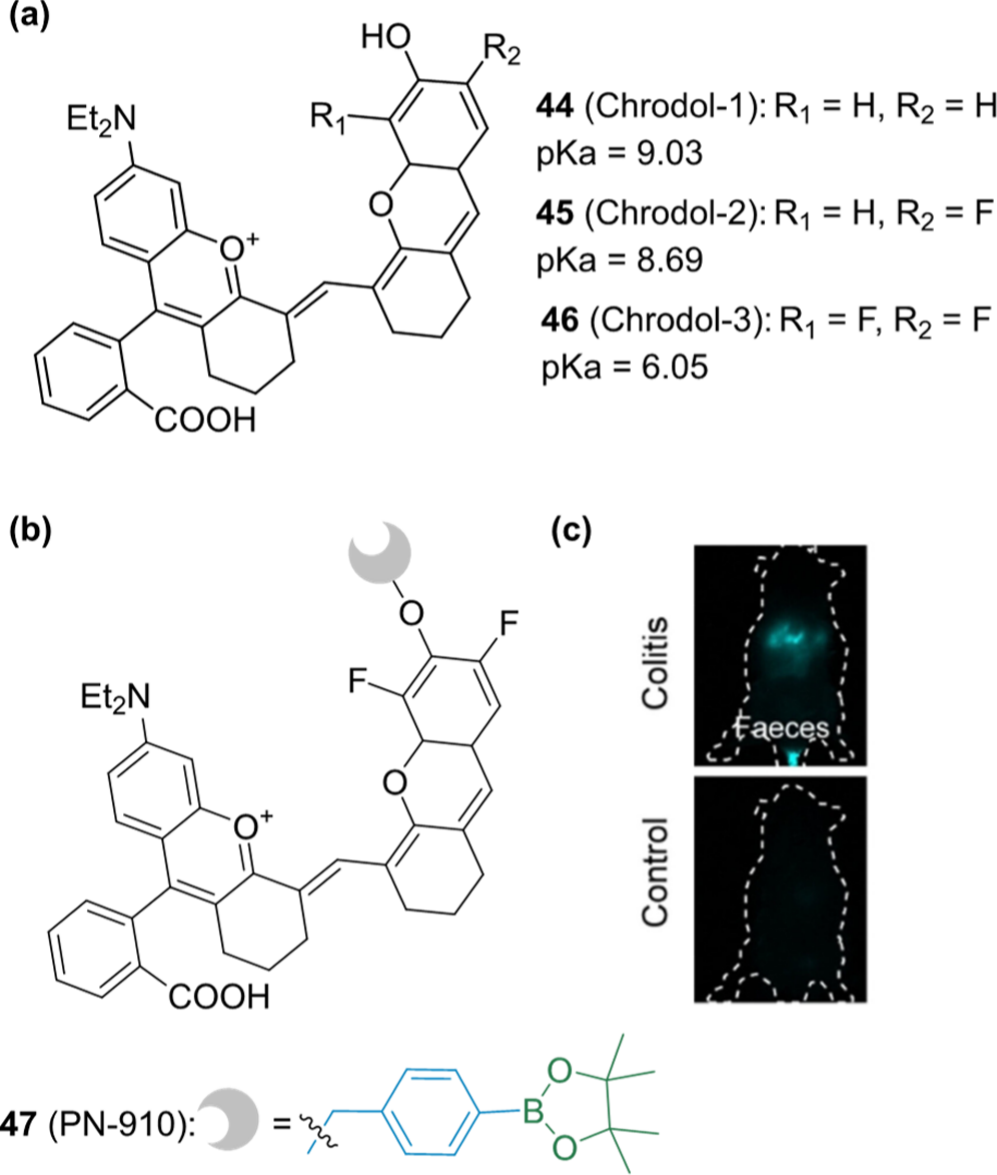
Merocyanine-based ABS sensors with tunable p*K*_a_. (a) Chemical structures of **44**, **45**, and **46** with varying fluoro-substitution. (b) Chemical structure of **47** (PN-910) with the boronic ester trigger for H_2_O_2_/Peroxynitrite detection. (c) In vivo fluorescence imaging of the colitis model versus control. Panel (c) Adapted with permission from ref 106 Copyright 2021 Wiley.

## 5 Conclusion and Outlook

The ability to visualize biological processes in their native context has motivated the development of numerous smart-sensing probes over the past two decades. Toward this end, activity-based fluorescent sensors have become an efficient methodology, where the modularity allows the separate development and combination of the responsive moieties for bioanalyte detection and the fluorophores that determine the photophysical performance of the probe. While the go-to fluorophore core for proof of concepts of new triggers may still remain as visible xanthene dyes such as coumarin, fluorescein, and rhodamine, which benefit from wide accessibility and facile functional groups for trigger attachment with efficient fluorescence on/off modulation, NIR and more recently SWIR fluorophore building blocks have emerged for developing biosensors in animal imaging experiments. We anticipate that the physiological relevance and clinical potential of detecting biological processes in animals will continue to drive the development of red-shifted fluorophores suitable for trigger attachment, advancing both biomedical research and clinical applications.

We have provided a brief overview of NIR and SWIR fluorophores that have been successfully incorporated into ABS sensors. The design principles of these fluorophores follow two routes, by extending the conjugation system of a visible fluorophore with a trigger attachment site, such as fluorescein, toward red-shifted wavelengths into NIR (e.g., BODIPY scaffolds), or by starting from an always-on NIR fluorophore and introduce a trigger attachment site with electronic insight into the on/off modulation (e.g., modified heptamethine dyes and hemicyanine scaffolds). In this context, a couple of fluorophore scaffolds hold promises for future repurposing into a fluorophore building block in ABS sensors. These may include the potential red-shifting of resorufin dyes and the attachment of triggers on the extended red-shifted xanthene dyes.^125–127^ The adaptation of spirocyclization on hybrid fluorophores^128–130^ and more recently on heptamethine dyes may also be incorporated into ABS sensors when the attachment sites are appropriately designed.^131–133^ Exposing the nitrogen atom of Cy7 dyes as a conjugation site provides another promising approach for ABS sensor integration,^134,135^ which can be made more generalizable, as it lifts the pH requirement of the fluorophore. Furthermore, integrating insights from across fluorophore families holds promise for developing new scaffolds with red-shifted wavelengths and convenient handles for trigger attachment. The maturation of NIR-II/SWIR building blocks for activity-based sensing will motivate researchers to adapt existing and new triggers for detection of biological events using these advantageous fluorophores. While early reports extensively use triggers for ROS as proof of concepts, we anticipate a rapid expansion to other biological analysts, including small signaling molecules, transition metals, and enzymatic activities, fulfilling the imaging toolbox for biological research and clinical diagnostics.

While being critical itself, being able to sense a biological event with a red-shifted dye has implications beyond simple turn-on fluorescence detection. Compared with visible wavelengths, the NIR/SWIR region offers a wider spectral space, making it possible to perform multiple imaging channels and follow different analytes at the same time to better understand how they interact in vivo. At the same time, ratiometric probes in the NIR/SWIR will benefit from its internal fluorescence reference, compensating for the complications in scattering and detection depth in animal applications. As these sensors move closer to applications with clinical relevance, attention will need to shift beyond optical performance to more practical issues, including solubility, stability, clearance, and safety, whether the probes are used as individual molecules or in formulated systems. This is especially important for extended conjugated scaffolds, which often show a tendency to aggregate or accumulate nonspecifically in biological environments. Ultimately, continued progress in ABS building block design should expand the range of biological processes that can be monitored in vivo and help move these technologies toward clinical use.

Moving beyond fluorescence detection, modular, red-shifted ABS building blocks can be combined with other modalities for multipurpose diagnostic agents. Through careful design, fluorescence ABS sensors can be complemented with other imaging modalities, such as photoacoustic imaging and magnetic resonance imaging,^135,136^ to improve accuracy and penetration depth. Furthermore, the free chromophores from ABS sensors feature an intense absorption in the NIR/SWIR region. This property may be leveraged in vivo to create smart actuators for precision treatment systems, such as light-directed drug delivery or phototherapy, where their activation by specific biological processes adds an additional layer of precision. Given the increasing interest in deciphering and manipulating biological activity in physiological contexts, we envision that new, enabling red-shifted fluorophores for ABS sensors will continue to be developed, which will greatly expand the capabilities of NIR/SWIR light-based biotechniques.

## Supplementary Material



## Notes

Photophysical properties, activation response, and biological applications of representative BODIPY-based fluorescent probes, heptamethine-based fluorescent probes, and hemicyanine-based fluorescent probes (PDF)
