# Supplementary material for: Near-Infrared and Shortwave Infrared Building Blocks for Activity-Based Sensors in Animals
Source: Bioconjug Chem. 2026 Jun 2;37(6):1089–107. doi: 10.1021/acs.bioconjchem.6c00039 (PMC13281388; doi:10.1021/acs.bioconjchem.6c00039)
Supplement: Supplementary file 1 [file bc6c00039_si_001.pdf]

Supporting Information for

Near-Infrared and Shortwave Infrared Building Blocks for Activity-Based Sensors in Animals

Tianmiao Wang,[†] *Xinyuan Li*[†]*, Lei Guo,*[‡] *and Shang Jia*[*†]

[†]Department of Chemistry and Biochemistry, Rutgers University - Newark, Newark, New Jersey 07102, United States

[‡]Department of Civil Engineering, University of Arkansas, Fayetteville, Arkansas 72701, United States

Table of Contents

Table S1. Photophysical Properties, Activation Response, and Biological Applications of Representative BODIPY-Based Fluorescent Probes. 3

Table S2. Photophysical Properties, Activation Response, and Biological Applications of Representative Heptamethine-Based Fluorescent Probes. 6

Table S3. Photophysical Properties, Activation Response, and Biological Applications of Representative Hemicyanine-Based Fluorescent Probes. 8

References 13

Table S1. Photophysical Properties, Activation Response, and Biological Applications of Representative BODIPY-Based Fluorescent Probes.

Probe	Trigger	Response	Abs$_{max}$ (OFF / ON, nm)	Em$_{max}$ (OFF / ON, nm)	Φ (OFF / ON, %)	ε_{max} (ON state, $M^{-1}\times cm^{-1}$)	Turn-On ratio	Biological Context
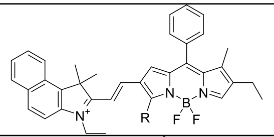	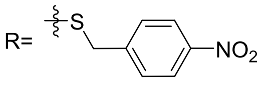 **1** NTR-InD[1]	NTR[a]	535 / 730	612 / 900	Low / 0.35	N.R.[b]	12×	Hypoxia-associated enzyme activity
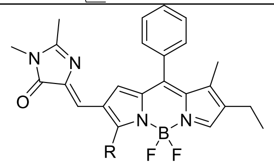	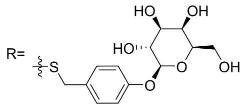 **2** BOD-K-βGal[2]	β-Gal[c]	533 / 670	565/715	Low / 0.39	8.0×10^4	N.R.	Ovarian cancer
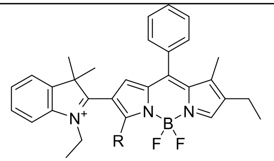	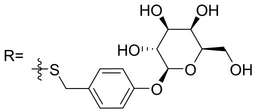 **3** BOD-M- βGal[2]	β-Gal	535 / 723	605 / 853	Low / 0.39	8.0×10^4	35×	Ovarian cancer
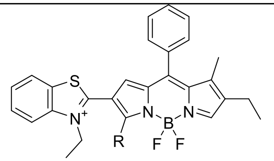	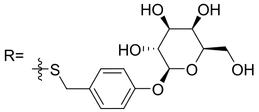 **4** BOD-II-NAG[3]	NAG[d]	490 / 710	N.R. / 1000	Low / 0.11	1.2×10^5	N.R.	Kidney injury
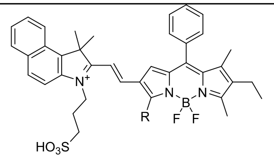 **5** WH-1[4]	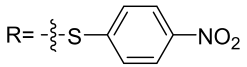	H_2S	521 / 762	521 / 762	521 / 762	N.R.	N.R.	Tumor imaging

**Probe**	**Trigger**	**Response**	**Abs_{max} (OFF / ON, nm)**	**Em_{max} (OFF / ON, nm)**	**Φ (OFF / ON, %)**	**$ε_{max}$ (ON state, $M^{-1}×cm^{-1}$)**	**Turn-On ratio**	**Biological Context**
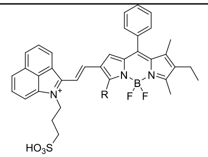 **6** WH-2[4]	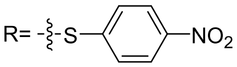	H_2S	561 / 850	561 / 850	N.R.	N.R.	10×	Tumor imaging
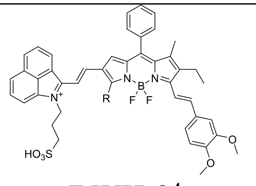 **7** WH-3[4]	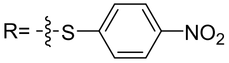	H_2S	575 / 925	680 / 1140	Low/ 0.17	N.R.	N.R.	Tumor imaging
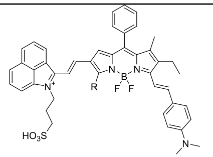 **8** WH-4[4]	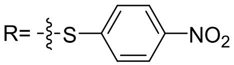	H_2S	645 / 960	761 / 1205	Low / 0.05	N.R.	N.R.	Tumor imaging
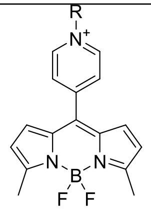	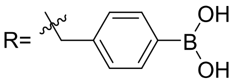 **9**[5]	ClO^-	507 / N.R.	N.R. / 515	0.002/ 0.213	N.R.	100×	Mitochondrial ClO^- imaging

Probe	Trigger	Response	Abs_{max} (OFF / ON, nm)	Em_{max} (OFF / ON, nm)	Φ (OFF / ON, %)	$ε_{max}$ (ON state, $M^{-1}×cm^{-1}$)	Turn-On ratio	Biological Context
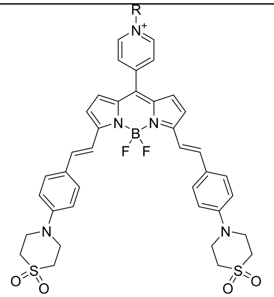	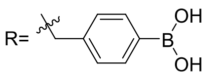 **10** FP-BDP4[6]	H_2O_2	689 / 670	725 / 720	Low / 0.21	$1.0×10^5$	30×	Cell and *in vivo* H_2O_2 imaging
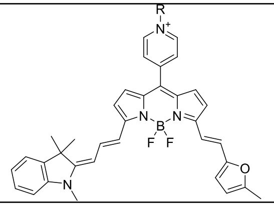	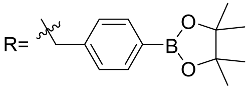 **11**[7]	$ONOO^-$	770 / 740	N.R. / 805	0.00019 / 0.36	N.R.	50×	NAFLD[e]

[a]NTR, Nitroreductase. [b]N.R., not reported. [c]β -Gal, β-Galactosidase. [d]NAG, N-acetyl-β-D-glucosaminidase. [e]NAFLD, Non-alcoholic fatty liver disease.

Table S2. Photophysical Properties, Activation Response, and Biological Applications of Representative Heptamethine-Based Fluorescent Probes.

Probe	Trigger	Response	Abs_{max} (OFF / ON, nm)	Em_{max} (OFF / ON, nm)	Φ (OFF / ON, %)	ε_{max} (ON state, $M^{-1}\times cm^{-1}$)	Turn-On ratio	Biological Context
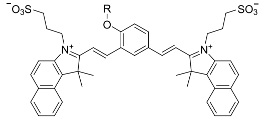	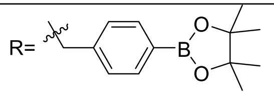 **13** Probe4[8]	H_2O_2	N.R.[a] / 590	N.R. / 720	N.R. / 0.16	5.22×10^4	10×	Inflammation
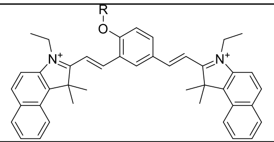	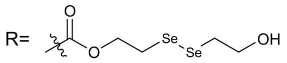 **14** BCy-SeSe[9]	GSH[b]	N.R. / 654	N.R. / 728	N.R.	N.R.	66×	Cerebral Ischemia–Reperfusion injury
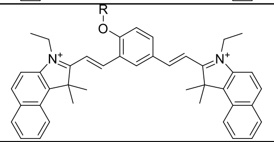	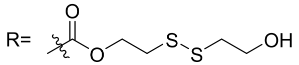 **15** BCy-SS[9]	GSH	N.R. / 654	N.R. / 728	N.R.	N.R.	N.R.	
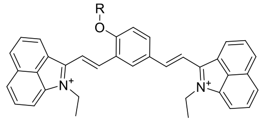	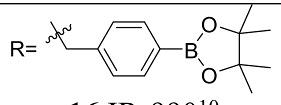 **16** IR-990[10]	H_2O_2	525 / 790	N.R. / 990	N.R.	N.R.	11.3×	DILI[c]
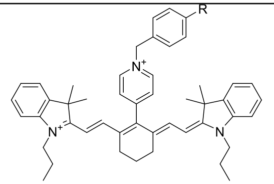	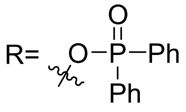 **17** CyP-DPP[11]	Superoxide	N.R. / 772	N.R. / 793	N.R. / 0.15	2.39×10^5	160×	Acute kidney injury

Probe	Trigger	Response	Abs_{max} (OFF / ON, nm)	Em_{max} (OFF / ON, nm)	Φ (OFF / ON, %)	$ε_{max}$ (ON state, $M^{-1}×cm^{-1}$)	Turn-On ratio	Biological Context
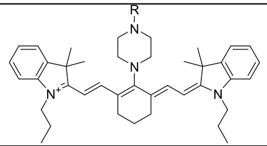	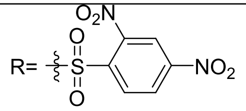 **18**[12]	GSH	N.R. / 730	N.R. / 736	N.R.	N.R.	N.R.	Cellular GSH
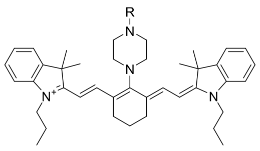	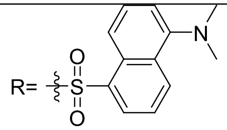 **19**[12]	GSH	N.R. / 730	N.R. / 736	N.R.	N.R.	N.R.	Cellular GSH
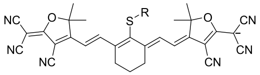	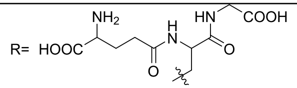 **20** CFC-GSH[13]	GGT[d]	890 / 650	930 / 660	N.R.	N.R.	N.R.	Hepatocellular carcinoma
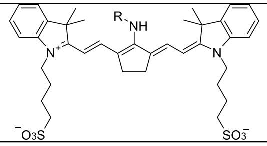	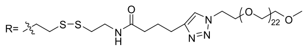 **21** Tg-PEG[14]	GSH	650 / 820	N.R. / 1000	N.R.	N.R.	50×	Tumor imaging

[a]N.R., not reported. [b]GSH, glutathione. [c]DILI, Drug-induced liver injury. [d]GGT, γ-glutamyl transpeptidase.

Table S3. Photophysical Properties, Activation Response, and Biological Applications of Representative Hemicyanine-Based Fluorescent Probes.

Probe	Trigger	Response	Abs_{max} (OFF / ON, nm)	Em_{max} (OFF / ON, nm)	Φ (OFF / ON, %)	ε_{max} (ON state, $M^{-1}\times cm^{-1}$)	Turn-On ratio	Biological Context
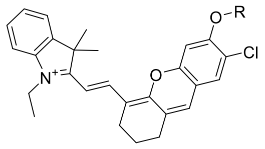	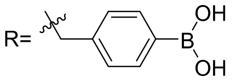 **22** NIR-H_2O_2[15]	H_2O_2	650 / 690	677 / 716	0.007 / 0.36	7.9×10^4	Over 200×	In vivo imaging
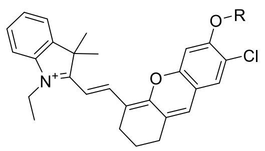	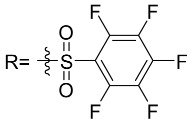 **23** HCy-H_2O_2[16]	H_2O_2	546 / 698	720 / 720	0.022 / 0.048	2.66×10^4	N.R.[a]	Mitochondrial oxidative stress
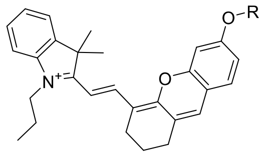	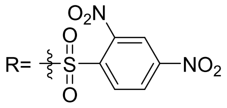 **24** HxpiS[17]	GSH[b]	590 / 681	N.R. / 703	0.0054 / 0.055	8.64×10^3	9.6×	Cellular biothiols
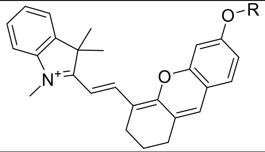	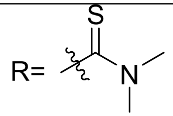 **25** CyClOP[18]	ClO^-	605 / 685	725 / 725	0.07 / 0.2	N.R.	15×	Endogenous HClO detection

Probe	Trigger	Response	Abs_{max} (OFF / ON, nm)	Em_{max} (OFF / ON, nm)	Φ (OFF / ON, %)	ε_{max} (ON state, $M^{-1}\times cm^{-1}$)	Turn-On ratio	Biological Context
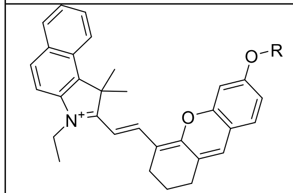	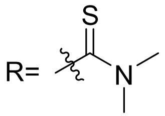 **26** NFL-S[19]	ClO^-	620 / 680	732 / 732	N.R.	N.R.	10×	Inflammation imaging
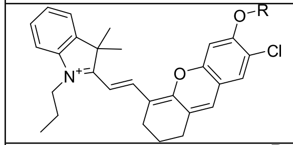	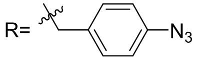 **27** NIR-Az[20]	H_2S	620 / 700	N.R. / 720	N.R.	N.R.	20×	Quantitative in vivo H_2S imaging
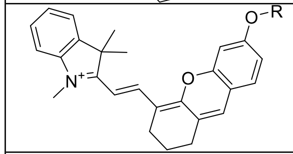	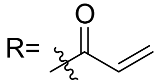 **28** CyA[21]	Cys[c]	582 / 674	696 / 697	0.016 / 0.51	5.5×10^4	10×	Fluorescence imaging of cellular Cys in HeLa cells
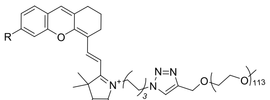	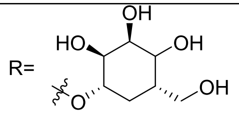 **29** CyGal-P[22]	β-Gal[d]	600 / 688	N.R. / 720	N.R.	N.R.	47×	Ovarian cancer cells (SKOV3) tumor-bearing mice imaging
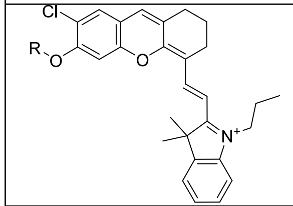	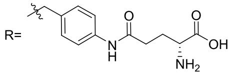 **30** GANP[23]	GGT[e]	563, 606 / 695	N.R. / 720	N.R. / 0.3	N.R.	100×	Non-invasively monitor GGT activity in living mice
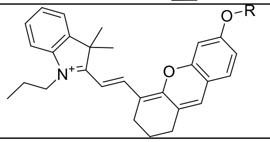	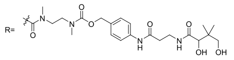 **31** CYLP[24]	Pantetheinase	590 / 680	N.R. / 710	<0.001 / 0.21	N.R.	40×	Inflammation imaging

Probe	Trigger	Response	Abs_{max} (OFF / ON, nm)	Em_{max} (OFF / ON, nm)	Φ (OFF / ON, %)	ε_{max} (ON state, $M^{-1}\times cm^{-1}$)	Turn-On ratio	Biological Context
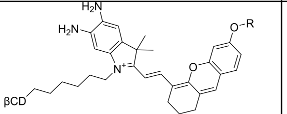	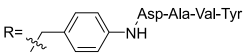 **32**[25]	Caspase-1, Nitric oxide	630 / 700	N.R. / 720	N.R.	N.R.	9×	Pulmonary inflammation imaging
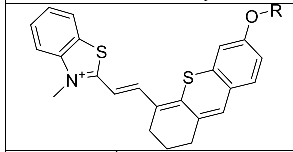	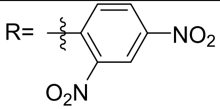 **33** HCy-HSP[26]	H_2S	565 / 720	N.R. / 787	N.R.	N.R.	52×	Acute lung injury imaging
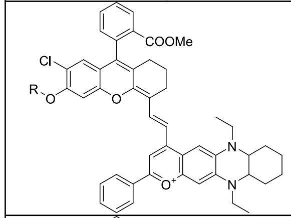	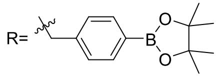 **34** NIRII-HD5-ONOO$^-$[27]	Peroxynitrite	723 / 854	N.R. / 895	N.R.	N.R.	18×	Lymphatic inflammation / tumor metastasis imaging
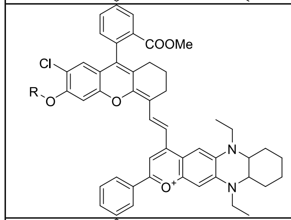	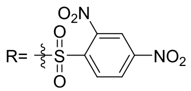 **35** NIRII-HD5-GSH[27]	GSH	723 / 854	N.R. / 895	N.R.	N.R.	22×	Lymphatic inflammation / tumor metastasis imaging
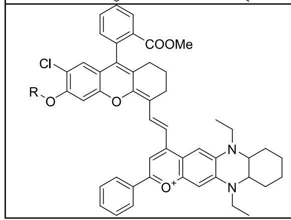	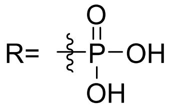 **36** NIRII-DH5-ALP[27]	Alkaline phosphatase	723 / 854	N.R. / 895	N.R.	N.R.	20×	Lymphatic inflammation / tumor metastasis imaging

Probe	Trigger	Response	Abs_{max} (OFF / ON, nm)	Em_{max} (OFF / ON, nm)	Φ (OFF / ON, %)	ε_{max} (ON state, $M^{-1}\times cm^{-1}$)	Turn-On ratio	Biological Context
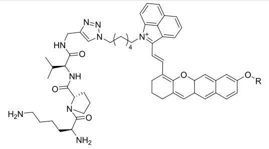	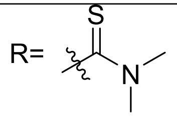 **37** HBC4[28]	ClO⁻	800	N.R. / 1088	N.R.	N.R.	N.R.	Inflammatory bowel disease imaging
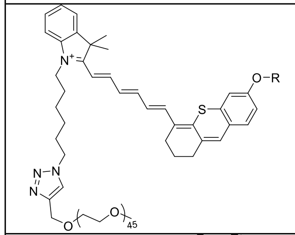	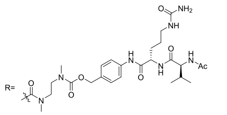 **38** SWIMP[29]	Cathepsin B	730 / 924	N.R. / 1010	N.R.	N.R.	12×	Lung metastasis imaging
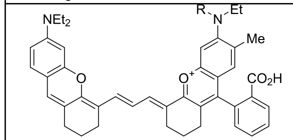	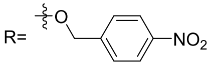 **40a** RAP-N[30]	Nitroreductase	805 / 965	940 / 1000	N.R.	N.R.	4×	Hypoxic tumor imaging
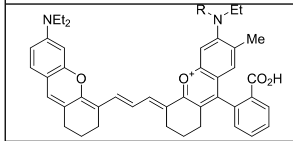	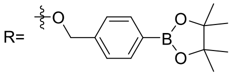 **40b** RAP-R[30]	Reactive oxygen species	805 / 965	940 / 1000	N.R.	N.R.	N.R.	Inflammation imaging
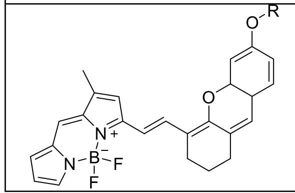	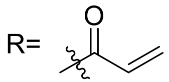 **42** BC-Cys[31]	Cys	710 / 800	N.R. / 905	N.R.	N.R.	65×	Liver injury / oxidative stress

Probe	Trigger	Response	Abs_{max} (OFF / ON, nm)	Em_{max} (OFF / ON, nm)	Φ (OFF / ON, %)	$ε_{max}$ (ON state, $M^{-1}×cm^{-1}$)	Turn-On ratio	Biological Context
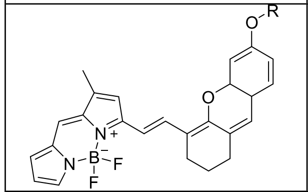	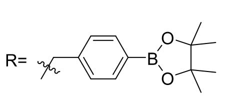 **43** BC-H_2O_2[31]	Liver injury / oxidative stress	710 / 840	N.R. / 905	N.R.	N.R.	43×	Drug-induced liver injury
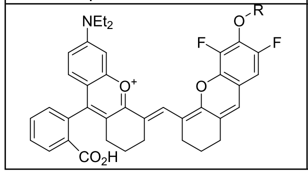	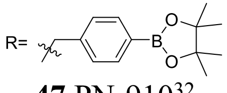 **47** PN-910[32]	Hydrogen peroxide / Peroxynitrite	675 / 870	N.R. / 908	N.R. / 0.34	N.R. / $1.2×10^5$	20×	Cystitis / Colitis

[a]N.R., not reported. [b]GSH, glutathione. [c]Cys, Cysteine. [d]β-Gal, β-Galactosidase. [e]GGT, γ-glutamyl transpeptidase

References

(1) Wang, R.; Chen, J.; Gao, J.; Chen, J.-A.; Xu, G.; Zhu, T.; Gu, X.; Guo, Z.; Zhu, W.-H.; Zhao, C. A Molecular Design Strategy toward Enzyme-Activated Probes with near-Infrared I and II Fluorescence for Targeted Cancer Imaging. *Chem Sci* **2019**, *10* (30), 7222–7227. https://doi.org/10.1039/C9SC02093D.

(2) Chen, J.-A.; Pan, H.; Wang, Z.; Gao, J.; Tan, J.; Ouyang, Z.; Guo, W.; Gu, X. Imaging of Ovarian Cancers Using Enzyme Activatable Probes with Second Near-Infrared Window Emission. *Chem Commun* **2020**, *56* (18), 2731–2734. https://doi.org/10.1039/C9CC09158K.

(3) Tan, J.; Yin, K.; Ouyang, Z.; Wang, R.; Pan, H.; Wang, Z.; Zhao, C.; Guo, W.; Gu, X. Real-Time Monitoring Renal Impairment Due to Drug-Induced AKI and Diabetes-Caused CKD Using an NAG-Activatable NIR-II Nanoprobe. *Anal Chem* **2021**, *93* (48), 16158–16165. https://doi.org/10.1021/acs.analchem.1c03926.

(4) Dou, K.; Feng, W.; Fan, C.; Cao, Y.; Xiang, Y.; Liu, Z. Flexible Designing Strategy to Construct Activatable NIR-II Fluorescent Probes with Emission Maxima beyond 1200 Nm. *Anal Chem* **2021**, *93* (8), 4006–4014. https://doi.org/10.1021/acs.analchem.0c04990.

(5) Li, G.; Ji, D.; Zhang, S.; Li, J.; Li, C.; Qiao, R. A Mitochondria-Targeting Fluorescence Turn-on Probe for Hypochlorite and Its Applications for in Vivo Imaging. *Sens Actuators B Chem* **2017**, *252*, 127–133. https://doi.org/10.1016/j.snb.2017.05.138.

(6) Lv, X.; Han, T.; Wu, Y.; Zhang, B.; Guo, W. Improving the Fluorescence Brightness of Distyryl Bodipys by Inhibiting the Twisted Intramolecular Charge Transfer Excited State. *Chem Commun* **2021**, *57* (76), 9744–9747. https://doi.org/10.1039/D1CC03360C.

(7) Tuo, Q.; Han, R.; Ma, X.; Wang, L.; Yang, K.; Wang, J.; Zhang, J. ONOO−-Activated Deep NIR-I Fluorescent Probe for Visualization of Non-Alcoholic Fatty Liver Disease. *Spectrochim Acta Mol Biomol Spectrosc* **2026**, *348*, 127097. https://doi.org/10.1016/j.saa.2025.127097.

(8) Karton-Lifshin, N.; Segal, E.; Omer, L.; Portnoy, M.; Satchi-Fainaro, R.; Shabat, D. A Unique Paradigm for a Turn-ON Near-Infrared Cyanine-Based Probe: Noninvasive Intravital Optical Imaging of Hydrogen Peroxide. *J Am Chem Soc* **2011**, *133* (28), 10960–10965. https://doi.org/10.1021/ja203145v.

(9) Zhang, X.; Huang, Y.; Han, X.; Wang, Y.; Zhang, L.; Chen, L. Evaluating the Protective Effects of Mitochondrial Glutathione on Cerebral Ischemia/Reperfusion Injury via Near-Infrared Fluorescence Imaging. *Anal Chem* **2019**, *91* (22), 14728–14736. https://doi.org/10.1021/acs.analchem.9b04082.

(10) Tian, Y.; Liu, S.; Cao, W.; Wu, P.; Chen, Z.; Xiong, H. H2O2-Activated NIR-II Fluorescent Probe with a Large Stokes Shift for High-Contrast Imaging in Drug-Induced Liver Injury Mice. *Anal Chem* **2022**, *94* (32), 11321–11328. https://doi.org/10.1021/acs.analchem.2c02052.

(11) Ouyang, J.; Sun, L.; Zeng, F.; Wu, S. Rational Design of Stable Heptamethine Cyanines and Development of a Biomarker-Activatable Probe for Detecting Acute Lung/Kidney Injuries via NIR-II Fluorescence Imaging. *Analyst* **2022**, *147* (3), 410–416. https://doi.org/10.1039/D1AN02183D.

(12) Yin, J.; Kwon, Y.; Kim, D.; Lee, D.; Kim, G.; Hu, Y.; Ryu, J.-H.; Yoon, J. Cyanine-Based Fluorescent Probe for Highly Selective Detection of Glutathione in Cell Cultures and Live Mouse Tissues. *J Am Chem Soc* **2014**, *136* (14), 5351–5358. https://doi.org/10.1021/ja412628z.

(13) Chu, F.; Feng, B.; Zhou, Y.; Liu, M.; Zhang, H.; Liu, M.; Chen, Q.; Zhang, S.; Ma, Y.; Dong, J.; Chen, F.; Zeng, W. Debut of Enzyme-Responsive Anionic Cyanine for Overlap-Free NIR-II-to-I Dual-Channel Tumour Imaging. *Chem Sci* **2025**, *16* (10), 4490–4500. https://doi.org/10.1039/D4SC06459C.

(14) Yin, L.; Xu, P.; Huang, Y.; Gu, X.; Sun, L.; Zhou, H.; Zhou, W.; Xie, C.; Fan, Q. Glutathione-Responsive Near-Infrared-II Fluorescence Probe for Early and Accurate Detection of In Situ and Metastatic Tumors. *Small* **2025**, *21* (30), 2503257. https://doi.org/10.1002/smll.202503257.

(15) Yuan, L.; Lin, W.; Zhao, S.; Gao, W.; Chen, B.; He, L.; Zhu, S. A Unique Approach to Development of Near-Infrared Fluorescent Sensors for in Vivo Imaging. *J Am Chem Soc* **2012**, *134* (32), 13510–13523. https://doi.org/10.1021/ja305802v.

(16) Zan, Q.; Zhao, K.; Li, R.; Yang, Y.; Yang, X.; Li, W.; Zhang, G.; Dong, C.; Shuang, S.; Fan, L. Mitochondria-Targetable Near-Infrared Fluorescent Probe for Visualization of Hydrogen Peroxide in Lung Injury, Liver Injury, and Tumor Models. *Anal Chem* **2024**, *96* (26), 10488–10495. https://doi.org/10.1021/acs.analchem.3c05479.

(17) Li, L.-L.; He, P.-Y.; Shi, L.; Pan, S.-L.; Li, M.-Y.; Zhou, Q.; Zhang, H.; Wang, N.; Li, K.; Yu, X.-Q. A Near-Infrared Water-Soluble Fluorescent Probe for the Detection of Biothiols in Living Cells and Escherichia Coli. *Anal Methods* **2019**, *11* (6), 821–826. https://doi.org/10.1039/C8AY02505C.

(18) Gao, W.; Ma, Y.; Liu, Y.; Ma, S.; Lin, W. Observation of Endogenous HClO in Living Mice with Inflammation, Tissue Injury and Bacterial Infection by a near-Infrared Fluorescent Probe. Sens. *Actuators B Chem* **2021**, *327*, 128884. https://doi.org/10.1016/j.snb.2020.128884.

(19) Qian, X.; Yu, H.; Zhu, W.; Yao, X.; Liu, W.; Yang, S.; Zhou, F.; Liu, Y. Near Infrared Fluorescent Probe for in Vivo Bioimaging of Endogenous Hypochlorous Acid. *Dyes Pigments* **2021**, *188*, 109218. https://doi.org/10.1016/j.dyepig.2021.109218.

(20) Park, C. S.; Ha, T. H.; Choi, S.-A.; Nguyen, D. N.; Noh, S.; Kwon, O. S.; Lee, C.-S.; Yoon, H. A Near-Infrared "Turn-on" Fluorescent Probe with a Self-Immolative Linker for the in Vivo Quantitative Detection and Imaging of Hydrogen Sulfide. *Biosens Bioelectron* **2017**, *89*, 919–926. https://doi.org/10.1016/j.bios.2016.09.093.

(21) Zhang, J.; Wang, J.; Liu, J.; Ning, L.; Zhu, X.; Yu, B.; Liu, X.; Yao, X.; Zhang, H. Near-Infrared and Naked-Eye Fluorescence Probe for Direct and Highly Selective Detection of Cysteine and Its Application in Living Cells. *Anal Chem* **2015**, *87* (9), 4856–4863. https://doi.org/10.1021/acs.analchem.5b00377.

(22) Zhen, X.; Zhang, J.; Huang, J.; Xie, C.; Miao, Q.; Pu, K. Macrotheranostic Probe with Disease-Activated Near-Infrared Fluorescence, Photoacoustic, and Photothermal Signals for Imaging-Guided Therapy. *Angew Chem Int Ed* **2018**, *57* (26), 7804–7808. https://doi.org/10.1002/anie.201803321.

(23) Luo, Z.; Feng, L.; An, R.; Duan, G.; Yan, R.; Shi, H.; He, J.; Zhou, Z.; Ji, C.; Chen, H.-Y.; Ye, D. Activatable Near-Infrared Probe for Fluorescence Imaging of γ-Glutamyl Transpeptidase in Tumor Cells and In Vivo. *Chem – Eur J* **2017**, *23* (59), 14778–14785. https://doi.org/10.1002/chem.201702210.

(24) Yang, Y.; Hu, Y.; Shi, W.; Ma, H. A Near-Infrared Fluorescence Probe for Imaging of Pantetheinase in Cells and Mice in Vivo. *Chem Sci* **2020**, *11* (47), 12802–12806. https://doi.org/10.1039/D0SC04537C.
(25) Hu, Y.; Liu, J.; Xu, M.; Pu, K. Dual-Locked Fluorescence Probe for Monitoring the Dynamic Transition of Pulmonary Macrophages. *J Am Chem Soc* **2025**, *147* (8), 7148–7157. https://doi.org/10.1021/jacs.5c00506.
(26) Su, W.; Huang, L.; Zhu, L.; Lin, W. A Novel Fluorescent Probe for Imagining Hydrogen Sulfide Upregulation in Acute Lung Injury. Sens. *Actuators B Chem* **2022**, *369*, 132297. https://doi.org/10.1016/j.snb.2022.132297.
(27) Qin, Z.; Ren, T.-B.; Zhou, H.; Zhang, X.; He, L.; Li, Z.; Zhang, X.-B.; Yuan, L. NIRII-HDs: A Versatile Platform for Developing Activatable NIR-II Fluorogenic Probes for Reliable In Vivo Analyte Sensing. *Angew Chem Int Ed* **2022**, *61* (19), 202201541. https://doi.org/10.1002/anie.202201541.
(28) Liu, Y.; Diao, S.; Ruan, B.; Zhou, Y.; Yu, M.; Dong, G.; Xu, W.; Ning, L.; Zhou, W.; Jiang, Y.; Xie, C.; Fan, Q.; Huang, J. Molecular Engineering of Activatable NIR-II Hemicyanine Reporters for Early Diagnosis and Prognostic Assessment of Inflammatory Bowel Disease. *ACS Nano* **2024**, *18* (11), 8437–8451. https://doi.org/10.1021/acsnano.3c13105.
(29) Yang, H.; Li, D.; Wu, J.; Pu, K. Shortwave Infrared Hemicyanine-6 for Cancer-Activated and Shaving-Free Preclinical Imaging of Lung Metastasis. *J Am Chem Soc* **2025**, *147* (34), 30794–30802. https://doi.org/10.1021/jacs.5c06682.
(30) Lan, Q.; Yu, P.; Yan, K.; Li, X.; Zhang, F.; Lei, Z. Polymethine Molecular Platform for Ratiometric Fluorescent Probes in the Second Near-Infrared Window. *J Am Chem Soc* **2022**, *144* (46), 21010–21015. https://doi.org/10.1021/jacs.2c10041.
(31) Zhang, L.; Yan, C.; Zhang, Y.; Ma, D.; Huang, J.; Zhao, Z.; Tao, Y.; Liu, C.; Li, J.; Zhu, W.-H.; Guo, Z. Activatable BODIPY-Chromene NIR-II Probes with Small Spectral Crosstalk Enable High-Contrast in Vivo Bioimaging. *Chem Commun* **2023**, *59* (54), 8388–8391. https://doi.org/10.1039/D3CC01742G.
(32) Zhang, X.; Chen, Y.; He, H.; Wang, S.; Lei, Z.; Zhang, F. ROS/RNS and Base Dual Activatable Merocyanine-Based NIR-II Fluorescent Molecular Probe for in Vivo Biosensing. *Angew Chem* **2021**, *133* (50), 26541–26545. https://doi.org/10.1002/ange.202109728.
